# *Aspergillus* Secondary Metabolite Database, a resource to understand the Secondary metabolome of *Aspergillus* genus

**DOI:** 10.1038/s41598-017-07436-w

**Published:** 2017-08-04

**Authors:** Varahalarao Vadlapudi, Nabajyoti Borah, Kanaka Raju Yellusani, Sriramya Gade, Prabhakar Reddy, Maheshwari Rajamanikyam, Lakshmi Narasimha Santosh Vempati, Satya Prakash Gubbala, Pankaj Chopra, Suryanarayana Murty Upadhyayula, Ramars Amanchy

**Affiliations:** 1 Pharmacology & Toxicology Division, CSIR-Indian Institute of Chemical Technology (IICT), Uppal Road, Tarnaka, Hyderabad 500 607 India; 2 National Institute of Pharmaceutical Education & Research (NIPER-Guwahati), 1st Floor, Institute of Pharmacy, Guwahati Medical College & Hospital, Narakasur Hill top, Guwahati, 781 032 India; 3 Division of Pharmacology & Toxicology, CSIR-IICT (Indian Institute of Chemical Technology), Ministry of Science & Technology (GOI), Uppal Road, Tarnaka, Hyderabad, Telangana 500017 India

## Abstract

*Aspergillus* is a genus of ubiquitous fungi that are pathologically & therapeutically important. Aspergillus Secondary Metabolites Database (A2MDB) is a curated compendium of information on *Aspergillus* & its secondary metabolome. A2MDB catalogs 807 unique non-redundantsecondary metabolites derived from 675 *Aspergillus* species. A2MDB has a compilation of 100 cellular targets of secondary metabolites, 44 secondary metabolic pathways, 150 electron and light microscopy images of various *Aspergillus* species. A phylogenetic representation of over 2500 strains has been provided. A2MDB presents a detailed chemical information of secondary metabolites and their mycotoxins. Molecular docking models of metabolite-target protein interactions have been put together. A2MDB also has epidemiological data representing Aspergillosis and global occurrence of *Aspergillus* species. Furthermore a novel classification of Aspergillosis along with 370 case reports with images, were made available. For each metabolite catalogued, external links to related databases have been provided. All this data is available on A2MDB, launched through Indian Institute of Chemical Technology, Hyderabad, India, as an open resource http://www.iictindia.org/A2MDB. We believe A2MDB is of practical relevance to the scientific community that is in pursuit of novel therapeutics.

## Introduction

The initial description and nomenclature of *Aspergillus* was credited to Micheli (1729), Haller (1768) and Fries (1832) who proposed a generic name for a group of fungi sharing similar morphological characteristics^1^. Microscopic examination reveals a spore bearing structure conidiophore, having aspergillum-like morphology. *Aspergillus* species thrive as endophytes^2^, saprophytes^3^, parasites^4^ and also as human pathogens^5–7^. *Aspergillus* species cause localized and systemic human conditions termed as Aspergilloses^8, 9^. They are also responsible for diseases in agricultural crops^10^ and domestic animals^10^.

Fungi are categorized and classified based on morphology and molecular genetics. Morphological characterization of various *Aspergillus* spp. requires a catalog of microscopic images and genetic examination of clinical strains needs the sequence information of ITS regions. *Aspergillus* species although ubiquitous are more frequently observed in immuno-compromised individuals upon inhalation of conidia. Most usual complications are lung and cutaneous infections. Over the last decade there has been an increase in Aspergillosis reports, an emerging infectious disease that can be fatal. There is a need for better understanding of Aspergillosis and a resource containing a collection of case reports and a fresh classification is very much necessary, as information is scattered all over the literature.

*Aspergillus* spp. produces a wide range of structurally heterogeneous secondary metabolites. that are of considerable interest to the scientific research community. Fungi of this genus produce important secondary metabolites that have industrial importance^11, 12^ and therapeutic significance like antibiotics^13^ and lovastatins^14^. As numerous natural products are being identified each day, a plethora of compounds still await discovery and a database can act as platform for their collection and annotation. Due to the need for *Aspergillus* centric metabolome repository, we have developed an open, user friendly resource; *A*2MDB that has experimental metabolomic data, catalogued and annotated with literature information. *A*2MDB provides an easy access to unbiased, comprehensive information about Aspergillosis, *Aspergillus* species, their secondary metabolites and cellular targets, molecular docking of metabolites–target interaction, secondary metabolic pathways, ITS based phylogeny and microscopic morphology. A2MDB also provides latest classification of Aspergillosis and collection of 370 case reports with over 70 reported variants of Aspergillosis. In the future more number of species, metabolite and molecular target data along with Aspergiloosis case reports will be included as and when additional information becomes available.

## A2MDB Database Development

### Data Mining

- Articles containing the search term *Aspergillus* (42189 articles as on 12/08/2016) and secondary metabolites (13770 articles as on 12/08/2016) were screened for cataloguing secondary metabolites from genus *Aspergillus* and this was collected with NCBI taxonomy ID and Mycobank ID.
- Microscopy images of *Aspergillus* species, Aspergillosis case reports and Secondary metabolite biosynthetic pathways were searched and collected from PubMed.
- ITS sequences of 7715 different *Aspergillus* species were collected from NCBI database for the primary analysis, out of which 2580 non-redundant sequences were from unique strains and species. The criterion for selection of a sequence was the availability of complete “18S-ITS1–5.8S-ITS2-28S” sequence. The non-redundant data was considered for the phylogenetic analysis.
- Multiple sequence alignment was performed using MAFFT 7^15^ and tree was constructed using UPGMA method. “iTOL” web interface was used for tree viewing and editing purpose^16^.
- All secondary metabolites archived in *A*2MDB have been linked to Public chemical compound databases^17^, PubChem^18^, ChemSpider^19^, TOXNET^20^, ChEBI^21^ and Chemical abstracts^22^. Classification of the 805 secondary metabolites has been done according to IUPAC nomenclature established and verified using NCBI-MeSH^23^.
- Tertiary structures of proteins described as cellular targets for the secondary metabolites were retrieved from Protein Data Bank (PDB). Protein Structures were optimized using Discovery Studio 4.5 Client^24^. The structures of secondary metabolites were downloaded from PubChem and optimized in Autodock^25^ and docked using AutoDock Vina^26^. The probable 3D models were generated using PyMOL^27^.
- *A*2MDB database technology: *A*2MDB database runs on MicroSoft.NET technology. ASP.NET and C#.net technologies have been used to build the dynamic web interface. C#, a server side scripting language, provides interface and assists in fetching data. ASP.NET Web pages function as HTML pages at run time. JavaScript was applied to ASP web pages for generating faster output with less stress on the server. *A*2MDB uses custom-designed lookup tables that ensure rapid responses to search queries. The relational architecture of *A*2MDB ensures data integrity and expandability, scope of the database. SQL server 2008 was used to facilitate back-end database support for storing the data and Asp.net as front end is used for fetching the data. Database has been provided a refined customized search functionality and search capability especially for the *Aspergillus* species, metabolites and docking images.
- Epidemiology: In order to visualize the global distribution of *Aspergillus* along with the Aspergillosis disease location, data mining was done from PubMed and based on the literature a global map was created using a R-package “rworldmap” (version 1.3–6)^28^ and spotting of the regions were done in Adobe Illustrator CC 2015, to showcase the incidence of *Aspergillus* spp. and Aspergillosis.

## Results and Discussion

**Figure 1:**
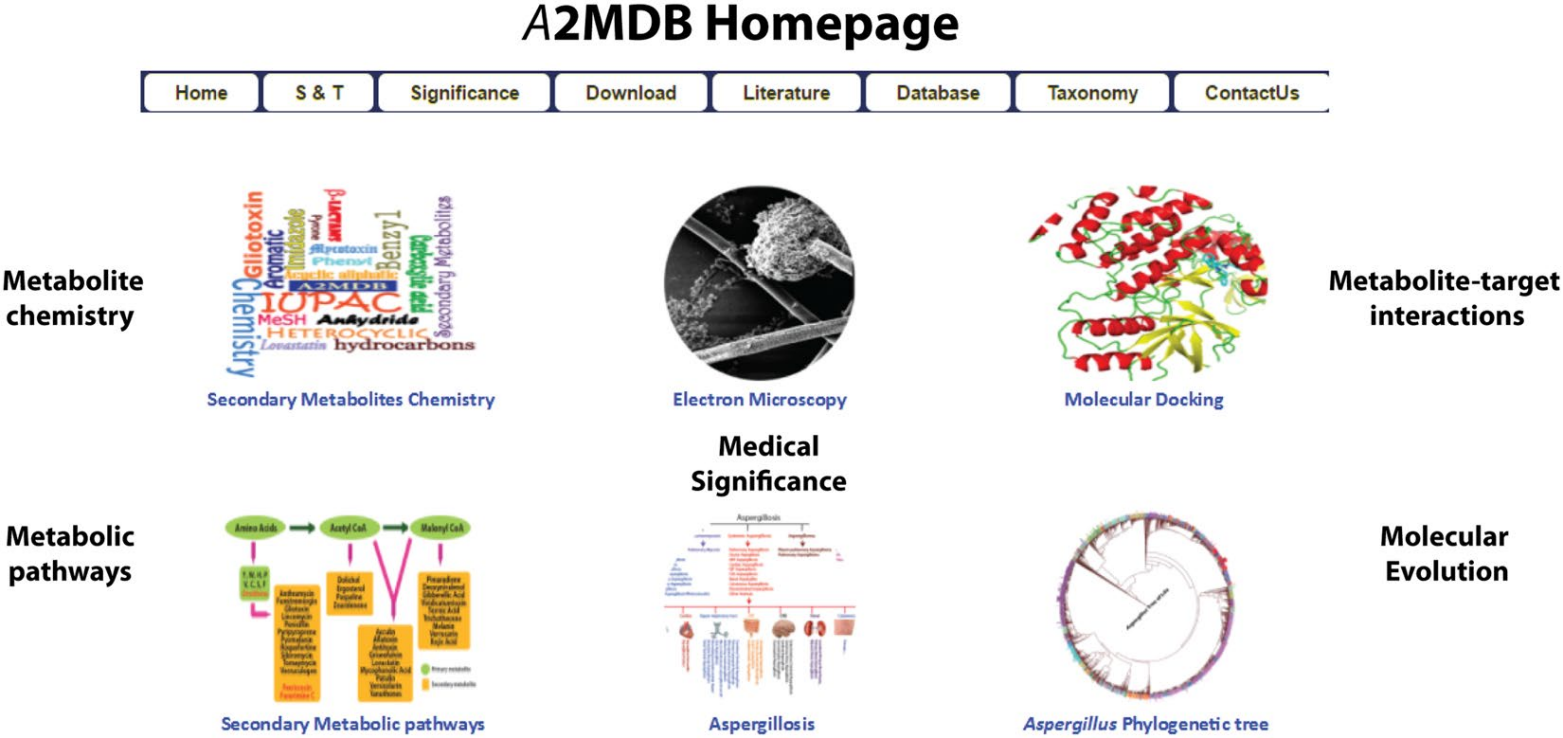
A screenshot of A2MDB showing multiple features in the homepage. The figure shows how clicking on various icons can navigate to the required option. Main page has icons for secondary metabolites chemistry, biological targets, Secondary metabolic pathways, microscopy images, molecular docking, Aspergillosis and phylogenetic analysis, apart from buttons which when clicked, explain science and significance of the project, database pages where one can surf and download data, taxonomy and contact us pages. The database has been officially launched through CSIR-IICT, India webpages.

*A*2MDB is a database that is organized around the central entity, *Aspergillus* genus focusing primarily on its Secondary Metabolites and their biological interactions with the basic goal of understanding metabolic pathways in *Aspergillus* spp. and Aspergillosis (Fig. 1). *A*2MDB is a one of a kind resource that provides access to unique secondary metabolites produced by *Aspergillus* species. *A*2MDB is an efficient, non-redundant, user-friendly resource for viewing, sorting and extracting information. Each set of data is connected to every other set of data so that every possible aspect related to species, metabolite, metabolic pathways and cellular targets available so far, is brought together and can be downloaded.

### A2MDB data retrieval, quality check and annotation

*A*2MDB is a curated secondary database to showcase metabolome of a group of fungi belonging to the genus *Aspergillus*. Information contained in it is, data related to *Aspergillus* ITS sequences, metabolites and their targets, electron microscopic images, information on Aspergillosis are based purely on text mining from PubMed and other authentic primary databases^17, 18, 29^.

Data entry in *A*2MDB follows a set protocol that enables (1) Identification of a new of species/strain with NCBI taxonomy ID, search and identification of any secondary metabolites with their chemical identifiers, (2) Collection of its genomic ITS data (3) search and identification of a metabolic target (4) metabolite-target interaction modeling (5) Search for a pathway (6) Search for microscopy information and further Aspergillosis components. In the Data curation of *A*2MDB, all the data has been verified by a curator besides the contributor and an additional scientist.

The quality of the data coming from a publication has already been peer reviewed and hence without bias full reference of the publication has been given for each and every piece of gathered data that has been provided. For quality check during curation of the data, \we generally follow a three step strategy.

1. Data collection: - The text mining is done only in the authenticated primary database and those data having valid reference are collected for entry in the A2MDB after being verified by 2 qualified scientists.
2. Pre-entry review: - In this step we follow the simple duplicate data entry validation system. For this purpose two independent files are produced for each dataset and these are compared to check for any discrepancy by two different scientists. After removing discrepancy and redundancy a final dataset is generated for entry to the database.
3. Post-entry review: - After the final tables were prepared, we check for any missing data and then a random sample of data is audited against the entered data to find manual errors.

The newer data coming in either by our group or externally will be entered after the three step cycle.

The data retrieval from primary databases although performed manually, was carried out with a systematic approach that dramatically reduced the work load in manual reviewing. We used the boolean and field operators in PubMed search^30^ to make our raw dataset as accurate as possible. Screening or pre-processing of the literature dataset was based on standard text mining procedure and after the data was collected it was annotated by scientists and uploaded and verified before making the database public.

### Searching and browsing A2MDB

*A*2MDB web application allows users to search or browse for *Aspergillus* Species resources by either specifying a search string or by choosing different optional filters to species name, metabolites, biological function, cellular target, PMID and Tax ID links. Search results are usually displayed as a tabular format and some browsable elements as PDF format. All the data available through A2MDB is downloadable and the content is available as tables as well as PDF files. *A*2MDB application also allows users to view all results on the same page at one time. The table consists of columns showing, for each resource, its species name, metabolites, biological function, cellular target, PMID and NCBI Taxonomy ID. In addition to providing comprehensive data, each metabolite and cellular target also contains hyperlinks to other authentic databases (PubChem, PubMed), references, digital images and applets for viewing directly from the primary database. Docking images are shown as image objects that show details of docking upon clicking the object.

### Metabolite information and classification

**Figure 2:**
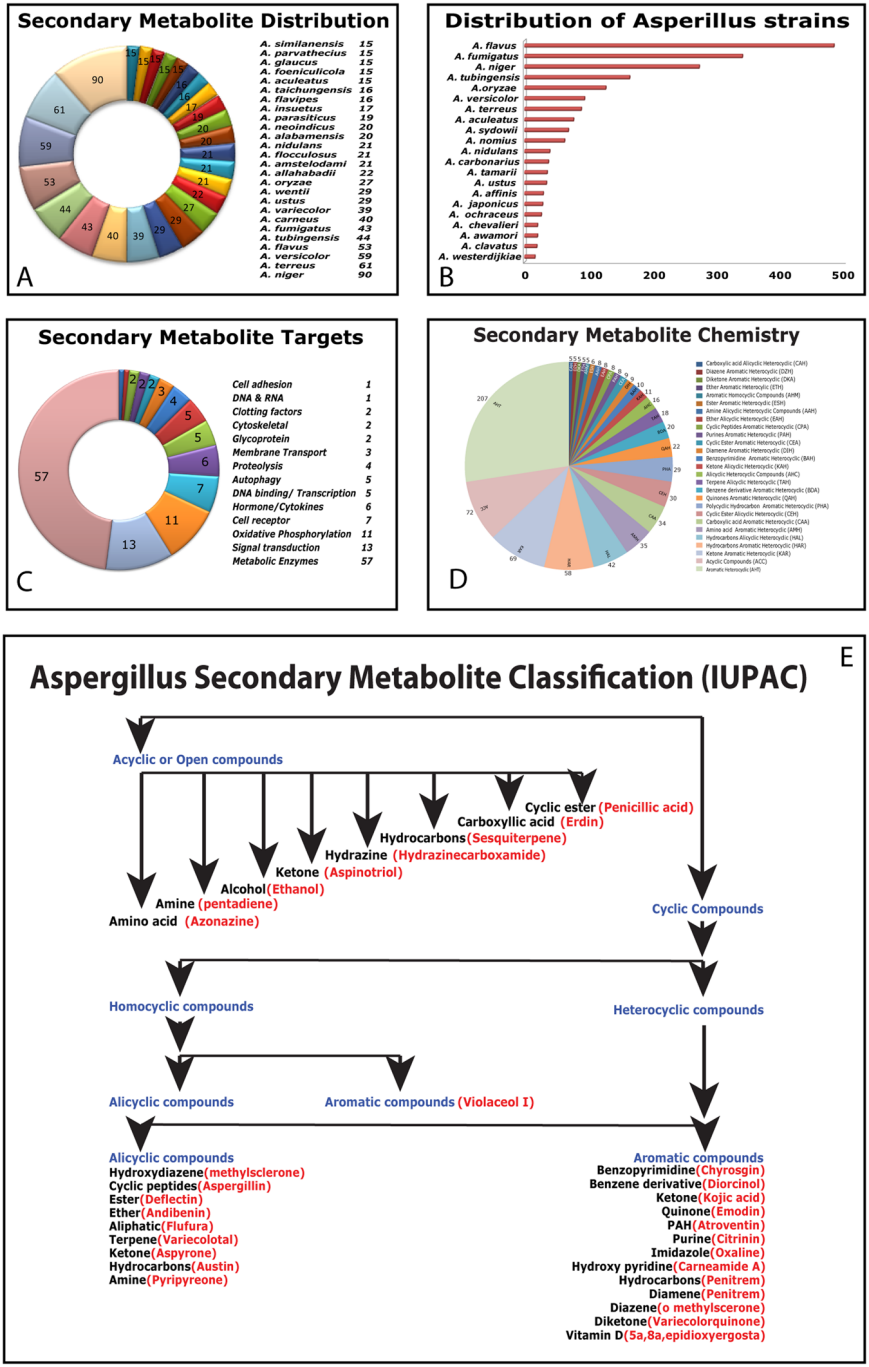
Analysis of the data collected for assimilation in to A2MDB: (**A**) pie chart representing the number of metabolites identified from major *Aspergillus* species, (**B**) bar graph shows the clinical distribution of biologically, medically and important strains of major *Aspergillus* species, (**C**) gene ontology based classification of cellular targets based on molecular function, (**D**) Distribution of secondary metabolites based on their chemical property, (**E**) IUPAC based classification of secondary metabolites in to various chemical compounds shown with examples under each category.

*A2MDB* has a collection of about 675 *Aspergillus* species, linked to a Taxonomy ID in NCBI database. 581 species have been annotated with links to Mycobank. 807 unique metabolites isolated from 324 species of this genus are incorporated so far with the objective of providing complete chemical and biological target related information. Nearly 25 species were identified for their variety of secondary metabolies produced. In an attempt to give complete chemical information, 523 of these secondary metabolites were connected to chemical databases like PubChem, ChemSpider, ChEBI, TOXNET. Among the secondary metabolites 213 were isolates from *A. flavus, A. fumigatus, A. oryzae and A. niger* of which 90 were from *A. niger* alone (Fig. 2A). This culmination among the highly enriched ones, is because these species are most studied owing to their etiological importance of some of the species.

Out of the 807 metabolites identified, majority of the secondary metabolites have been identified from around 25 *Aspergillus* species (Fig. 2A). 60 different Mycotoxins were identified (Supplementary Table 1) from 35 species of *Aspergillus* that pose a considerable amount of threat to veterinary and human health^31^. Aflatoxins produced by *Aspergillus flavus* and *Aspergillus parasiticus* have been known to be carcinogenic and hepatotoxic in nature. Aflatoxin B1 the most toxic mycotoxin can penetrate through the skin^32^. Ochratoxin, contaminant of cereals, is primarily produced by *Aspergillus ochraceus* ^33^ causes liver damage, enterititis, immunosuppression, teratogenesis, nephrotoxicity and renal tumors^31^ Citrinin is a polyketide mycotoxin, produced by *Aspergillus candidus* and *Aspergillus carneus* shows nephrotoxic, hepatotoxic and cyototoxic behaviour. Another lethal mycotoxin sterigmatocystin is mainly found in dairy products, is a possible carcinogen mainly produced by *Aspergillus nidulans* and *Aspergillus versicolor*. Patulin is a mycotoxin produced by *Aspergillus giganteus* usually associated with spoilage of apple and grapes. causes cerebral hemorrhage. Within each species, several biologically important strains; were reported that are either pathogenic, or that are industrially or biologically important (Fig. 2B). A vast majority of strains that are clinically, biolocically and industrially important were identified to be from 18 species (Fig. 2B) which not only shows the infectious nature of these species in producing toxins but also of their industrial importance by the nature of the important secondary metabolites produced. We observed that majority of these secondary metabolites had been found to be targeting metabolic enzymes in order to manipulate cellular machinery (Fig. 2C). These metabolites have been linked to a unique registry number provided by Chemical Abstracts Service (CAS) of the American Chemical Society^22^ and classified into categories based on IUPAC nomenclature (Fig. 2D,E). Whole genome sequencing of *Aspergillus* species has pointed out to a varying diversity of the enzymes involved in the secondary metabolism and a range of novel compounds remain elusive and uncharacterized^34^. Analysis is ongoing to identify secondary metabolism gene clusters in over 250 species of *Aspergillus*.

### Secondary metabolic pathways

**Figure 3:**
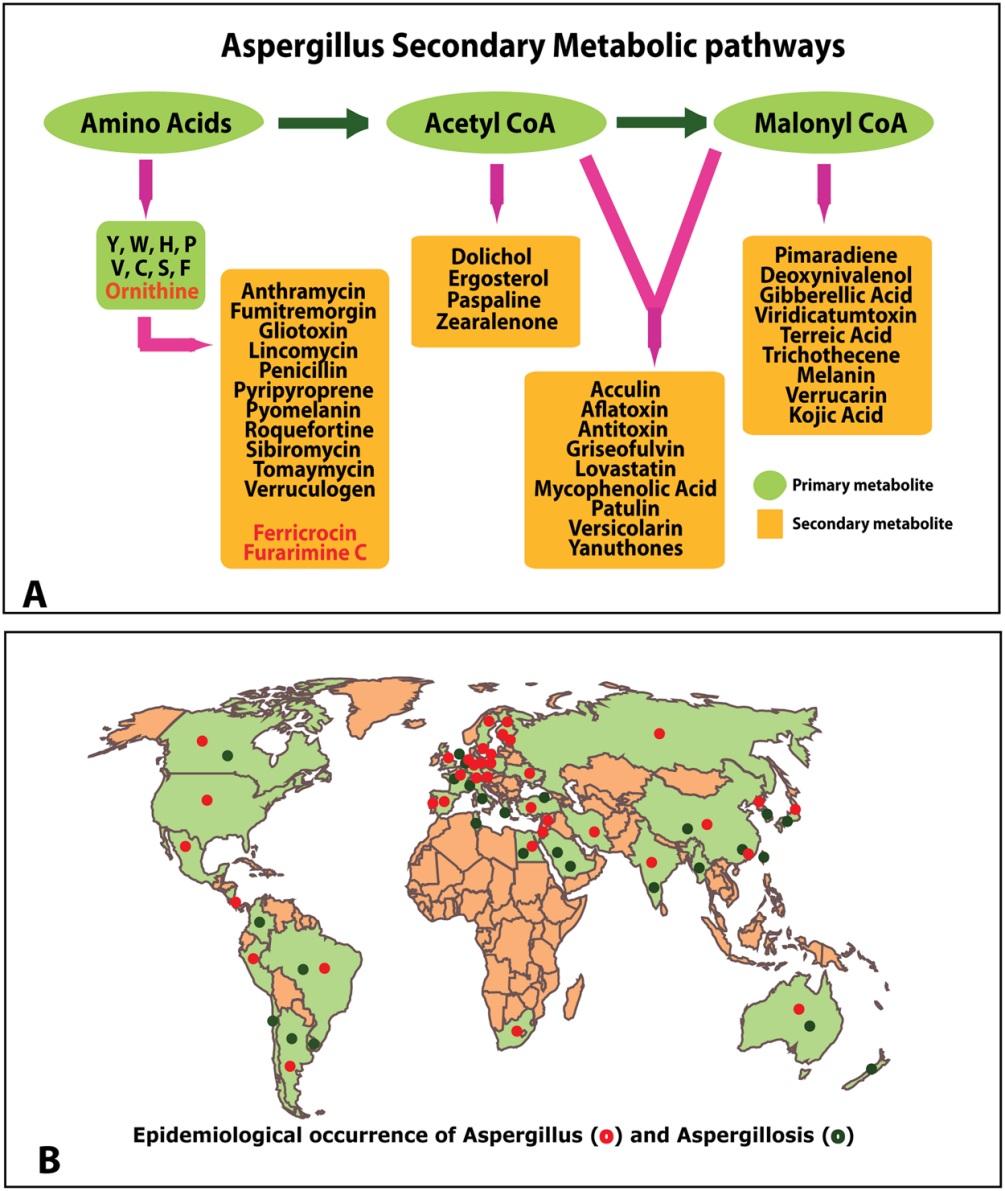
(**A**) A representation of metabolic pathways where in the primary metabolic intermediates serve as precursors for secondary metabolite production in *Aspergillus*, (**B**) A qualitative epidemiological distribution map was created using a R-package “rworldmap” (version 1.3–6) to showcase the incidence of *Aspergillus* spp. and Aspergillosis.

Primary metabolism is well studied in fungi and well documented in many databases and secondary metabolites and metabolic pathways in fungi are underrepresented in databases. Precursors derived from the primary metabolic pathways are siphoned into secondary metabolic pathways to synthesize compounds that have unusual structures and biological properties (Figs 2D and 3A). There is an increasing need to understand secondary metabolism to exploit these organisms, and control the production of potential drugs and toxins. In *A*2MDB we provide 44 secondary metabolic pathway illustrations directly taken from the literature with references. 133 biological targets for 135 of these metabolites have been identified from the literature so far. We have also furnished external links to 1578 metabolic pathways from KEGG database that are *Aspergillus* specific (1447 primary and 131 secondary metabolic pathways). We have also provided information about anti-fungal compounds and their bioactivity on *Aspergillus* species, with relevant information from ChEMBL^35^.

### Epidemiology

As *Aspergillus* produces small hydrophobic conidia that easily disperse into air and can survive in the drastic environmental condition, distribution of *Aspergillus* species is found to be ubiquitous across different ecological niche^3^. The geographical map was provided that provides clear correlation of the occurrence of the pathogen and disease (Fig. 3B).

**Figure 4:**
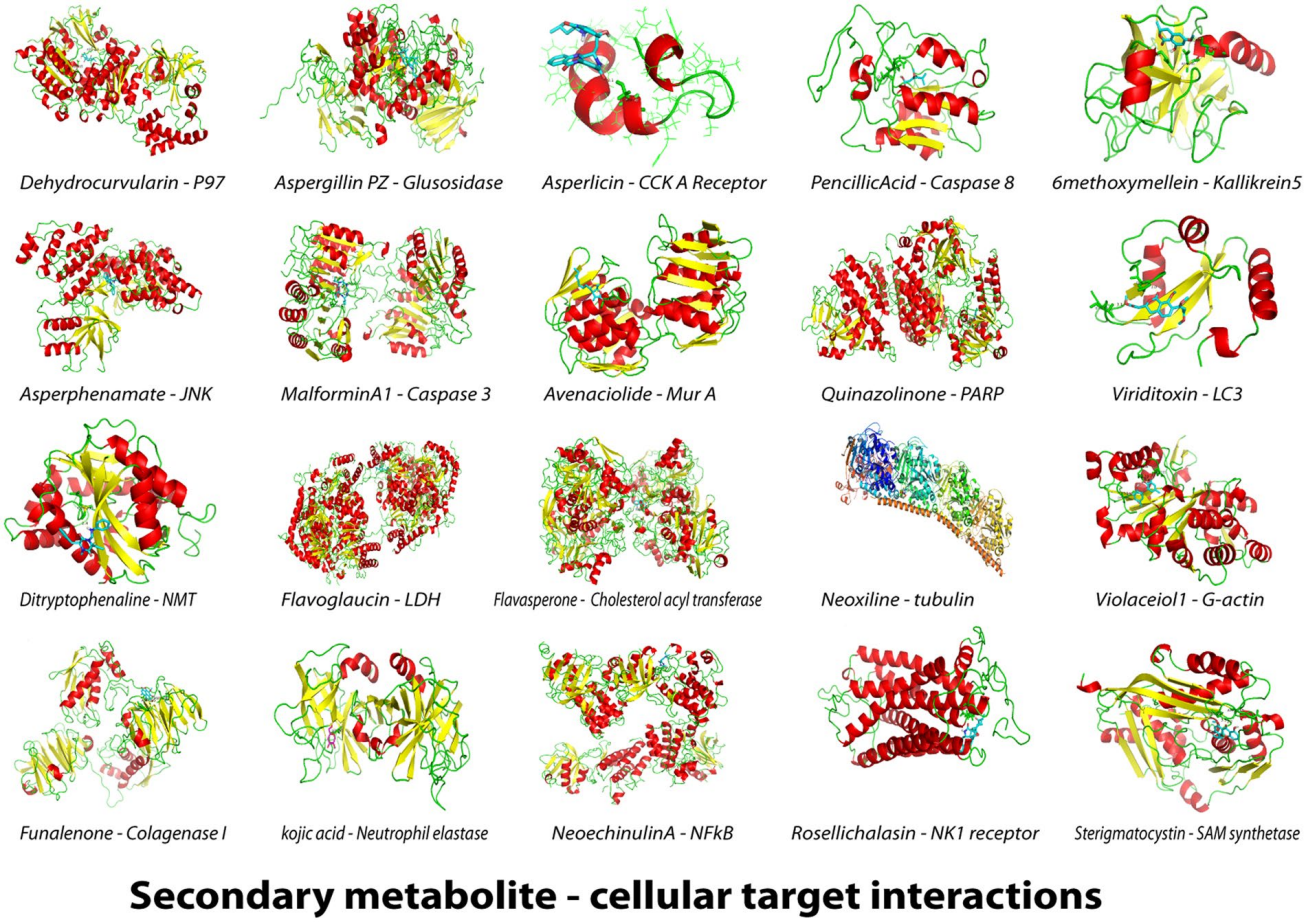
Molecular docking models representing secondary metabolite and cellular target interactions as reported in literature. Docking was performed using Auto Dock Vena. The protein structure was from PDB, metabolite structure from one of the chemical databases as explained in the text and binding energies are also represented.

Molecular modelling of secondary metabolite - target interactions were provided to further validate their likely metabolite-target interactions with PDB structures based on docking energies and binding affinities (Fig. 4). Further studies are being carried out to understand their structure activity relationships.

### Molecular and Microscopic examination

In *A*2MDB we provide 223 Microscopy images of *Aspergillus* spp.; a collection of literature derived electron and light microscopy images is provided with links to original published articles. Morphological information by means of optical and electron microscopy images as well as molecular phylogenetic information by means of ITS sequences as reported earlier was provided.

### Aspergillosis classification and Case studies

**Figure 5:**
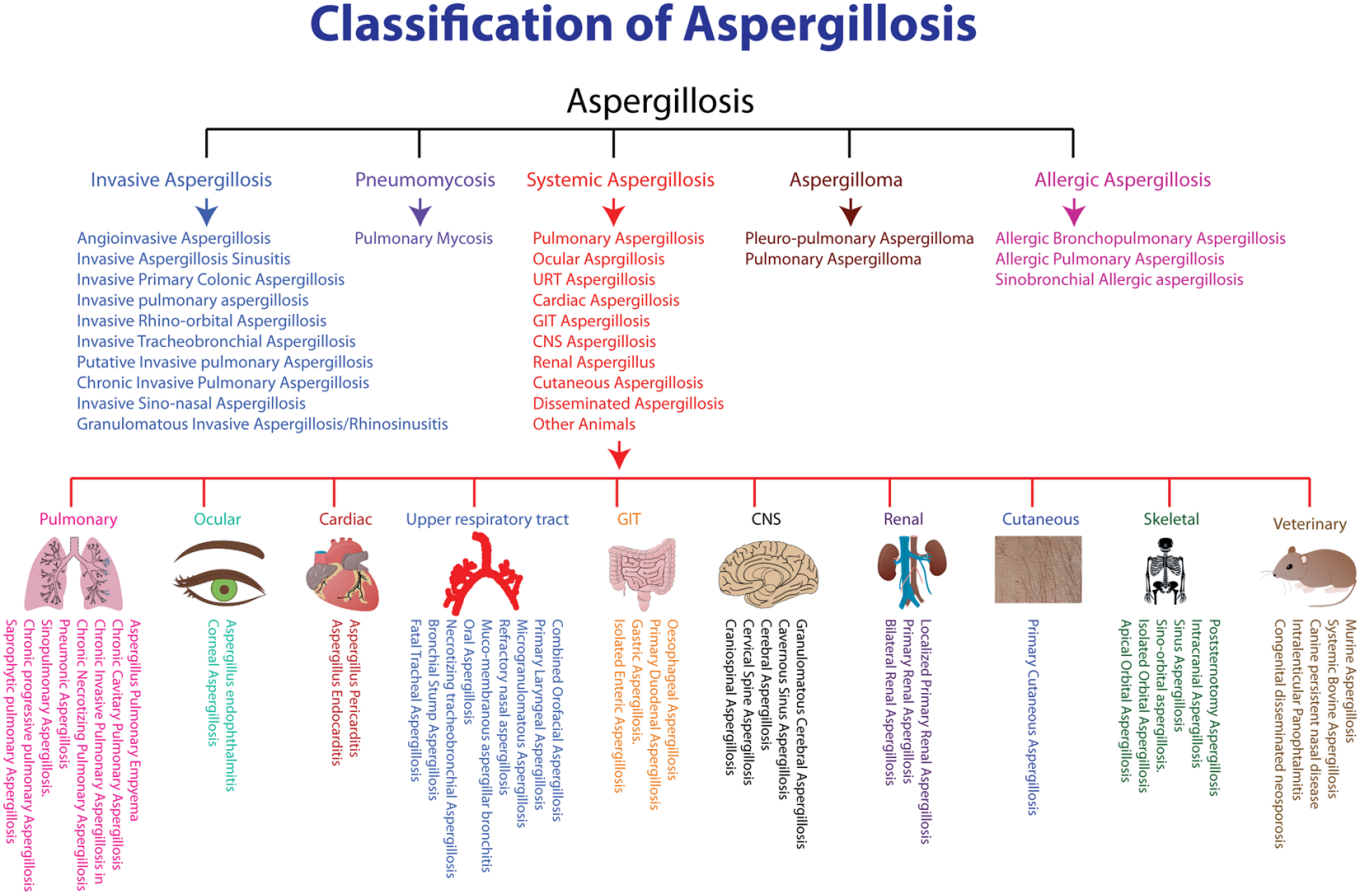
A detailed classification of Aspergillosis with case reports and related images from the literature available so far from humans and animals with DOI literature references. All the organ systems have been drawn using the software Edraw (Version 8.6) trial version.

Aspergillosis caused by various *Aspergillus* spp. has been classified broadly in to 5 different categories and over 70 different types (Fig. 5) that fall under 5 primary categories (invasive aspergillosis, pneumomycosis, systemic aspergillosis, aspergilloma and allergic aspergillosis. Aspergillosis in humans and animals has been mentioned in depth using case reports and CT scan images wherever available from 370 PubMed articles each one available for download with article identifier.

### Taxonomical information

**Figure 6:**
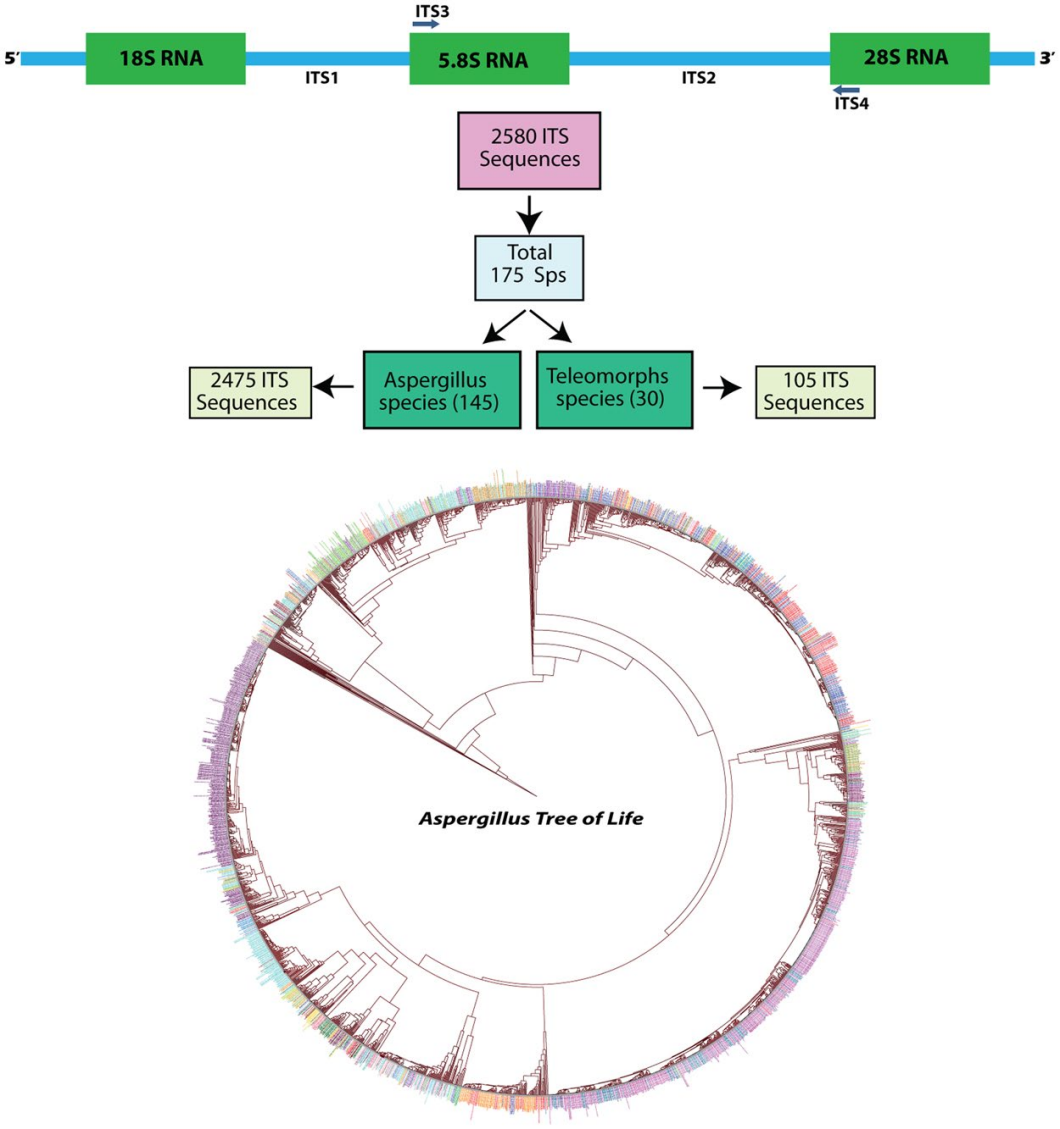
Phylogenetic tree of life for 2850 *Aspergillus* strains using ITS sequences. All the sequences are unique, various strains of *Aspergillus* belong mainly to 175 species.

A phylogenetic tree of life based on 2580 ITS sequences, selected from 175 unique strains and species of *Aspergillus* (Fig. 6) was represented. A significant number of *Aspergillus* species can be associated with 11 different sexual stages (teleomorphs); *Emericella*, *Eurotium*, *Fennellia*, *Neosartorya*, *Petromyces*, *Dichotomomyces, Chaetosartorya, Cristaspora, Penicilliopsis, Sclerocleista* and *Phialosimplex* that have now come under the umbrella of *Aspergillus* after the decision of “one name one fungi” by International Code of Nomenclature. U.S. Department of Energy, Joint Genome Institute (DOE-JGI) has about 270 *Aspergillus* species/strains/teleomorphs in their genome sequencing list, out of which nearly 90 have been sequenced and the others in either sequencing pipeline or in review as part of 1000 fungal genome project.

#### Comparison with other fungal and metabolic databases

*A*2MDB is a non-redundant comprehensive compilation of *Aspergillus* specific secondary metabolism information. There are few databases that provide species specific information and even fewer on secondary metabolism and secondary metabolites of biological importance. While databases like PHI-base^36^, EHFPI^37^, catalogue genetic information to aide pathogenicity studies, ITS2^38^, metaMicrobesOnline^39^, Mycobank^29^ provide for phylogenetic and taxonomic information. Primary metabolic pathways in eukaryotes are well annotated by databases like KEGG^40^, Reactome^41^ and HMDB^42^, while Metacyc, Biocyc and Humancyc are pathway genome databases^43, 44^. Metacyc provides a list of natural compounds but is not organism specific. AntiSMASH^45^ and SMURF^46^ are web applications that predict bacterial and fungal secondary metabolic gene clusters^45^. Databases like YEASTRACT^47^, PomBase^48^, NetwoRx^49^ are specific to *Saccharomyces* species, but not to secondary metabolite/metabolism information. ‘*Aspergillus* and Aspergillosis’ resource provides secondary metabolite information from *Aspergillus* species, but redundancy exists and sources remain undefined with major focus on aspergillosis^50^ and is currently merged with Central *Aspergillus* Data REpository (CADRE)^51^. C*A*DRE is a repository of genomic data of *Aspergillus* species^51^. *Aspergillus* Genome Database (AspGD) is a genetic information resource specific to *Aspergillus* spp.^52^. However, *A2MDB* is a unique database that provides comprehensive secondary metabolite information related to vast number of *Aspergillus* species with resourceful evidence based information pertaining to Aspergillosis, *Aspergillus* specific anti-fungal compounds, ATCC *Aspergillus* collection, secondary metabolic pathways, phylogeny and other related databases. We believe that *A2MDB* will find a global niche with its unique content.

#### Future Directions

1. *A*2MDB has documented 807 secondary metabolites and almost 500 of these have chemical information from established chemical databases and we are trying to provide hyperlinks for the rest of the compounds to well established chemical and metabolite databases. Search for new metabolites is being actively done from the 351 species, that do not have prior reports on secondary metabolite isolation.
2. *A*2MDB has 370 case reports with images from 70 different types of Aspergillosis that was classified afresh. We are also trying to find information about *Aspergillus* causing diseases in plants.
3. Since some of the species are endophytes we are trying to identify and categorize *Aspergillus* species as endophytes and relationship with their host plants.
4. More species are being added along with their electron and light microscopy images as and when they become available.
5. Addition of transformation products of *Aspergillus* and derivatives of its secondary metabolites is under progress as well as their pathway annotation.
6. Molecular docking studies are being attempted using QSAR that might result in discovery of new therapeutic compounds.
7. Analysis is ongoing on identification of secondary metabolism gene clusters in all of the genome sequenced *Aspergillus* species.
8. Identification of primary metabolite that becomes the parent compound for the secondary metabolite is also being carried out.

## Conclusions

*A*2MDB is a unique non-redundant resource of secondary metabolites and pathways information dedicated to *Aspergillus* species and Aspergillosis*. A*2MDB is regularly updated by database administrators and scientists. We welcome community participation in depositing and sharing the data. *A*2MDB is available for free without any registration. We believe *A*2MDB will be of immense importance to mycologists as well as scientists looking for important natural products obtained from *Aspergillus* species.

### Data Accessibility

All the data being reported in this manuscript that has been collected, curated and deposited has been made publicly available directly through our database in the database download options (http://www.iictindia.org/a2mdb).

## Electronic supplementary material

Supplementary Information
