# Supplementary material for: Aspergillus Secondary Metabolite Database, a resource to understand the Secondary metabolome of Aspergillus genus
Source: Sci Rep. 2017 Aug 4;7:7325. doi: 10.1038/s41598-017-07436-w (PMC5544713; doi:10.1038/s41598-017-07436-w)
Supplement: Supplementary file 1 — Supplementary Information [file 41598_2017_7436_MOESM1_ESM.pdf]

Supplementary Information for the manuscript SREP-17-11102:

***Manuscript Title**: Aspergillus* Secondary Metabolite Database, a resource to understand the Secondary metabolome of *Aspergillus* genus

Authors: Varahalarao Vadlapudi[§#], Nabajyoti Borah[§#], Kanaka Raju Yellusani[§#], Sriramya Gade[§], Prabhakar Reddy[§], Maheshwari Rajamanikyam[§], Lakshmi Narasimha Santosh Vempati[§], Satya Prakash Gubbala[§], Pankaj Chopra [§♦], Suryanarayana Murty Upadhyayula*[♦], Ramars Amanchy*

Supplementary Table 1		
Aspergillus Secondary Metabolites Classification based on IUPAC		
Mycotoxins are highlighted in green		
Secondary Metabolite	MeSH Nomenclature	IUPAC Classification
Ethanol	Acyclic Alchol	Alchol
N-benzoyl-L-phenylalaninol	Acyclic Alchol	Ethanolamines
1,4-pentadiene	Acyclic Amine	Pyridines
AcT1	Acyclic Amino acid	Peptides
Aculin A	Acyclic Amino acid	Peptides
Aspercryptin	Acyclic Amino acid	Oligopeptides
Aspergillomarasmine B	Acyclic Amino acid	Aspartic acid
Aspergillomarasmine A	Acyclic Amino acid	Aspartic acid
Aspergillus acid A	Acyclic Amino acid	Aspartic acid
Aspergillus acid C	Acyclic Amino acid	Aspartic acid
Aspergillus acid D	Acyclic Amino acid	Aspartic acid
Azonazine	Acyclic Amino acid	Dipeptides
Bicoumanigrin	Acyclic Amino acid	Dipeptides
Cryptoechinuline D	Acyclic Amino acid	Alanine
Cryptoechinuline E	Acyclic Amino acid	Alanine
Cryptoechinuline F	Acyclic Amino acid	Alanine
Cryptoechinuline B	Acyclic Amino acid	Alanine
Cryptoechinuline C	Acyclic Amino acid	Alanine
Cryptoechinuline G	Acyclic Amino acid	Alanine
Cycloaspeptide B	Acyclic Amino acid	Peptides
Cycloaspeptide C	Acyclic Amino acid	Peptides
Cycloaspeptide A	Acyclic Amino acid	Peptides
Desferritriacetylfusigen	Acyclic Amino acid	Ornithine
Desoxyverrucarin E	Acyclic Amino acid	Ornithine
Echinocandin B	Acyclic Amino acid	Peptides
Emerin	Acyclic Amino acid	Thymopoetins
Hydrophobin	Acyclic Amino acid	Fungal Protein
Hydroxymethylsterigmatocystin	Acyclic Amino acid	Fungal Protein
Miyakamide	Acyclic Amino acid	Peptides
Miyakamide A1	Acyclic Amino acid	Peptides
Miyakamide A2	Acyclic Amino acid	Peptides
Miyakamide B1	Acyclic Amino acid	Peptides
Miyakamide B2	Acyclic Amino acid	Peptides
Oryzamide A	Acyclic Amino acid	Peptides
Restrictocin	Acyclic Amino acid	Fungal Proteins
Scleramide	Acyclic Amino acid	Peptides
Terrelumamide A	Acyclic Amino acid	Peptides
Terrelumamide B	Acyclic Amino acid	Peptides
Dihydromevinolin	Acyclic Amino acid	Oxidoreductase
Hydrazinecarboxamide	Acyclic Hydrazine	Hydrazine
Sesquiterpene	Acyclic Hydrocarbons	Terpene
Dihydroaspyrone	Acyclic Ketone	Pyrones

Aspinotriol	Acyclic Ketone	Polyketones
Aspinotriol B	Acyclic Ketone	Polyketones
Cis-3,6-dibenzyl-3,6-bis(methylthio) piperazine-2,5-dione	Alicyclic Heterocyclic	Piperazine
Citraconic anhydride	Alicyclic Heterocyclic	Furan
Flufuran	Alicyclic Heterocyclic	Furan
Asperteretal A	Alicyclic Heterocyclic	Hydroxyfuran
Asperteretal B	Alicyclic Heterocyclic	Hydroxyfuran
Asperteretal C	Alicyclic Heterocyclic	Hydroxyfuran
Dehydrocarolic acid	Alicyclic Heterocyclic	Furans
Carnemycin A	Alicyclic Heterocyclic	Indole alkaloids
Carnemycin B	Alicyclic Heterocyclic	Indole alkaloids
Dihydrouridine	Alicyclic Heterocyclic	Pyrimidine
Nigragillin	Alicyclic Heterocyclic	Alkaloid
Acetylaranotin	Alicyclic Heterocyclic	Oxepins
Tenuazonic acid	Alicyclic Heterocyclic	Pyrollidines
Asperopterin B	Alicyclic Heterocyclic	Pyrimidine
Asperopterin A	Alicyclic Heterocyclic	Dihydroxypteridine
13-O-Methylviriditin	Alicyclic Heterocyclic	Pyrrolidne
Pyripyropene A	Alicyclic Heterocyclic Amine	Pyridines
Pyripyropene B	Alicyclic Heterocyclic Amine	Pyridines
Pyripyropene C	Alicyclic Heterocyclic Amine	Pyridines
Pyripyropene D	Alicyclic Heterocyclic Amine	Pyridines
Pyripyropene E	Alicyclic Heterocyclic Amine	Pyridines
Pyripyropene E	Alicyclic Heterocyclic Amine	Pyridines
Pyripyropene S	Alicyclic Heterocyclic Amine	Pyridines
Pyripyropene T	Alicyclic Heterocyclic Amine	Pyridines
Terreic acid	Alicyclic Heterocyclic Diketone	Quinones
22-Deacetylyanuthone A	Alicyclic Heterocyclic Hydrocarbons	Terpene
2-Hydroxydiplopterol	Alicyclic Heterocyclic Hydrocarbons	Terpene
6-epi-ophiobolin K	Alicyclic Heterocyclic Hydrocarbons	Sesterterpenes
6-methoxy-5(6)-dihydropenicillic acid	Alicyclic Heterocyclic Hydrocarbons	Terpene
Austin	Alicyclic Heterocyclic Hydrocarbons	Terpene
Austinol	Alicyclic Heterocyclic Hydrocarbons	Terpene
Dehydroaustinol	Alicyclic Heterocyclic Hydrocarbons	Terpene
Dehydroaverufin	Alicyclic Heterocyclic Hydrocarbons	Terpene
Ochraceopone A	Alicyclic Heterocyclic Hydrocarbons	Sesquiterpene
Ochraceopone B	Alicyclic Heterocyclic Hydrocarbons	Sesquiterpene
Ochraceopone C	Alicyclic Heterocyclic Hydrocarbons	Sesquiterpene
Ochraceopone D	Alicyclic Heterocyclic Hydrocarbons	Sesquiterpene
Ochraceopone E	Alicyclic Heterocyclic Hydrocarbons	Sesquiterpene
Ophiobolin	Alicyclic Heterocyclic Hydrocarbons	Sesterterpenes
Ophiobolin A	Alicyclic Heterocyclic	Sesterterpenes

	Hydrocarbons	
Ophiobolin K	Alicyclic Heterocyclic Hydrocarbons	Sesterterpenes
Ophiobolins	Alicyclic Heterocyclic Hydrocarbons	Sesterterpenes
Parasiticolide	Alicyclic Heterocyclic Hydrocarbons	Terpene
Parasiticolide A	Alicyclic Heterocyclic Hydrocarbons	Terpene
Variecolin	Alicyclic Heterocyclic Hydrocarbons	Terpene
Variecolorin A	Alicyclic Heterocyclic Hydrocarbons	Terpene
Variecolorin B	Alicyclic Heterocyclic Hydrocarbons	Terpene
Variecolorin C	Alicyclic Heterocyclic Hydrocarbons	Terpene
Variecolorin D	Alicyclic Heterocyclic Hydrocarbons	Terpene
Variecolorin E	Alicyclic Heterocyclic Hydrocarbons	Terpene
Variecolorin F	Alicyclic Heterocyclic Hydrocarbons	Terpene
Variecolorin G	Alicyclic Heterocyclic Hydrocarbons	Terpene
Variecolorin H	Alicyclic Heterocyclic Hydrocarbons	Terpene
Variecolorin I	Alicyclic Heterocyclic Hydrocarbons	Terpene
Variecolorin J	Alicyclic Heterocyclic Hydrocarbons	Terpene
Variecolorin K	Alicyclic Heterocyclic Hydrocarbons	Terpene
Variecolorin L	Alicyclic Heterocyclic Hydrocarbons	Terpene
Yanuthone A	Alicyclic Heterocyclic Hydrocarbons	Terpene
Yanuthone B	Alicyclic Heterocyclic Hydrocarbons	Terpene
Yanuthone C	Alicyclic Heterocyclic Hydrocarbons	Terpene
Yanuthone D	Alicyclic Heterocyclic Hydrocarbons	Terpene
Yanuthone E	Alicyclic Heterocyclic Hydrocarbons	Terpene
**6β**-methoxyergosta-7,22-diene-**3β,5α**-diol	Alicyclic Heterocyclic Hydrocarbons	Steroid
Oxylipin	Alicyclic Heterocyclic Hydrocarbons	Fatty acids
Dehydroaustin	Alicyclic Heterocyclic Hydrocarbons	Polyketides
Epiaspinonediol	Alicyclic Heterocyclic Hydrocarbons	Polyketides
Sequoiamonascin	Alicyclic Heterocyclic Hydrocarbons	Polyketides
Aspyrone	Alicyclic Heterocyclic Ketone	Pyrones
Aspyronol	Alicyclic Heterocyclic Ketone	Pyrones
Asteltoxin	Alicyclic Heterocyclic Ketone	Pyrones
Asteltoxin B	Alicyclic Heterocyclic Ketone	Pyrones
Decaturin	Alicyclic Heterocyclic Ketone	Pyrones
Kojic acid	Alicyclic Heterocyclic Ketone	Pyrones
Maltol	Alicyclic Heterocyclic Ketone	Pyrones

Mellamide	Alicyclic Heterocyclic Ketone	Pyrones
Decumbenone A	Alicyclic Heterocyclic Ketone	Ketones
Decumbenone B	Alicyclic Heterocyclic Ketone	Ketones
Decumbenone C	Alicyclic Heterocyclic Ketone	Ketones
1'-O-ethyl-6,8-di-O-methylaverantin	Alicyclic Heterocyclic Quinone	Anthraquinones
2-Methyl-1,4-benzoquinone 5,6-epoxide	Alicyclic Heterocyclic Quinone	Benzo quinone
Helvolic acid	Alicyclic Heterocyclic Terpene	Fusidic acid
(E)-6-(4'-hydroxy-2'-butenoyl)-strobilactone A	Alicyclic Heterocyclic Terpene	Sesquiterpene
14-deacetyl parasiticolide A	Alicyclic Heterocyclic Terpene	Sesquiterpene
**2α, 9α, 11**-trihydroxy-6-oxodrim-7-ene	Alicyclic Heterocyclic Terpene	Terpenoids
Andilesin	Alicyclic Heterocyclic Terpene	Terpenoids
Andilesin A	Alicyclic Heterocyclic Terpene	Terpenoids
Andilesin B	Alicyclic Heterocyclic Terpene	Terpenoids
Andilesin C	Alicyclic Heterocyclic Terpene	Terpenoids
Anditomin	Alicyclic Heterocyclic Terpene	Terpenoids
Dihydrotubingensin B	Alicyclic Heterocyclic Terpene	Indole diterpene
Dihydrotubingensin A	Alicyclic Heterocyclic Terpene	Indole Terpen
Emindole SC	Alicyclic Heterocyclic Terpene	Alkaloids
Insuetolide A	Alicyclic Heterocyclic Terpene	Terpenoids
Insuetolide B	Alicyclic Heterocyclic Terpene	Terpenoids
Insuetolide C	Alicyclic Heterocyclic Terpene	Terpenoids
Strobilactone A	Alicyclic Heterocyclic Terpene	Meroterpenoids
Tetranorlabdane diterpenoid	Alicyclic Heterocyclic Terpene	Diterpene
Variecolactol	Alicyclic Heterocyclic Terpene	Sesterterpene
Arestrictin A	Alicylic Heterocyclic Diamene	Piperazines
Arestrictin B	Alicylic Heterocyclic Diamene	Piperazines
Quadrilineatin	Aromatic Heterocyclic	Aldehyde
4-aryl-quinolin-2-one	Aromatic Heterocyclic	Quinolines
4-Methylprimaquine	Aromatic Heterocyclic	Quinolines
Atromentin	Aromatic Heterocyclic	Indole alkaloids
Brevianamide K	Aromatic Heterocyclic	Indole alkaloids
Brevianamide M	Aromatic Heterocyclic	Indole alkaloids
Fumigaclavine A	Aromatic Heterocyclic	Indole alkaloids
Fumigaclavine B	Aromatic Heterocyclic	Indole alkaloids
Fumigaclavine C	Aromatic Heterocyclic	Indole alkaloids
Fumigatoside A	Aromatic Heterocyclic	Indole alkaloids
Fumigatoside B	Aromatic Heterocyclic	Indole alkaloids
Fumigatoside C	Aromatic Heterocyclic	Indole alkaloids
Fumigatoside D	Aromatic Heterocyclic	Indole alkaloids
Isotryptoquivaline	Aromatic Heterocyclic	Quinazolinones
Neoechinuline	Aromatic Heterocyclic	Indole
Norisotryptoquivaline	Aromatic Heterocyclic	Indole
Okaramin	Aromatic Heterocyclic	Indole alkaloids
Okaramine A	Aromatic Heterocyclic	Indole alkaloids
Okaramine B	Aromatic Heterocyclic	Indole alkaloids
Okaramine H	Aromatic Heterocyclic	Indole alkaloids
O-methyl sterigmatocystin	Aromatic Heterocyclic	Xanthanes
O-Methyldihydrosterigmatocystin	Aromatic Heterocyclic	Xanthanes
Physcion anthrone B	Aromatic Heterocyclic	Heterocyclic aromatic
Rubrofusarin B	Aromatic Heterocyclic	Benzofurans
Rubrofusarin B	Aromatic Heterocyclic	Benzofurans

Serantrypinone	Aromatic Heterocyclic	Indole alkaloids
Taichunamide A	Aromatic Heterocyclic	Indole alkaloids
Taichunamide B	Aromatic Heterocyclic	Indole alkaloids
Taichunamide C	Aromatic Heterocyclic	Indole alkaloids
Taichunamide D	Aromatic Heterocyclic	Indole alkaloids
Taichunamide E	Aromatic Heterocyclic	Indole alkaloids
Taichunamide F	Aromatic Heterocyclic	Indole alkaloids
Taichunamide G	Aromatic Heterocyclic	Indole alkaloids
Tubingensin A	Aromatic Heterocyclic	Alkaloids
Tubingensin B	Aromatic Heterocyclic	Alkaloids
Versicone A	Aromatic Heterocyclic	Alkaloids
Versicone E	Aromatic Heterocyclic	Alkaloids
Versicone F	Aromatic Heterocyclic	Alkaloids
Versicone G	Aromatic Heterocyclic	Alkaloids
Versicone H	Aromatic Heterocyclic	Alkaloids
Indol alkaloid	Aromatic Heterocyclic	Benzopyrrole
Indole	Aromatic Heterocyclic	Benzopyrrole
Asperlicin	Aromatic Heterocyclic	Benzodiazepenes
Asperlin	Aromatic Heterocyclic	Benzodiazepenes
Asperline	Aromatic Heterocyclic	Benzodiazepenes
Aspernigrin A	Aromatic Heterocyclic	Benzodiazepenes
Circumdatin A	Aromatic Heterocyclic	Benzodiazepenes
Circumdatin B	Aromatic Heterocyclic	Benzodiazepenes
Circumdatin J	Aromatic Heterocyclic	Benzodiazepenes
9-deacetoxyfumigaclavine C	Aromatic Heterocyclic	Indole
9-deacetylfumigaclavine C	Aromatic Heterocyclic	Indole
**9ξ**-O-2 Brevianamide Q	Aromatic Heterocyclic	Indole
Aflatrem	Aromatic Heterocyclic	Indole
Aflavinine	Aromatic Heterocyclic	Indole
Amauromine	Aromatic Heterocyclic	Indole
Asperazine	Aromatic Heterocyclic	Indole
Aspochalamin	Aromatic Heterocyclic	Indole
Aspochalasin A	Aromatic Heterocyclic	Indole
Aspochalasin B	Aromatic Heterocyclic	Indole
Aspochalasin D	Aromatic Heterocyclic	Indole
Aspochalasin F	Aromatic Heterocyclic	Indole
Aspochalasin G	Aromatic Heterocyclic	Indole
Aspochalasin H	Aromatic Heterocyclic	Indole
Aspochalasin I	Aromatic Heterocyclic	Indole
Aspochalasin J	Aromatic Heterocyclic	Indole
Aspochalasin K	Aromatic Heterocyclic	Indole
Asposterol	Aromatic Heterocyclic	Indole
Aspterric acid	Aromatic Heterocyclic	Indole
Asterriquinone	Aromatic Heterocyclic	Indole
Aszonalenin	Aromatic Heterocyclic	Indole
Austamide	Aromatic Heterocyclic	Indole
Cyclopiazonic Acid	Aromatic Heterocyclic	Indole
Cytochalasin E	Aromatic Heterocyclic	Indole
Cytochalasin Z24	Aromatic Heterocyclic	Indole
Cytochalasins	Aromatic Heterocyclic	Indole
D-Altritol	Aromatic Heterocyclic	Indole

Demethyl asterriquinone	Aromatic Heterocyclic	Indole
Demethylkotanin	Aromatic Heterocyclic	Indole
Dihydroxyaflavinine	Aromatic Heterocyclic	Indole
Diketopiperazine	Aromatic Heterocyclic	Indole
Gliotoxin	Aromatic Heterocyclic	Indole
Gliotoxin G (1)	Aromatic Heterocyclic	Indole
Gliotoxin G (2)	Aromatic Heterocyclic	Indole
Notoamide S	Aromatic Heterocyclic	indole
Nygerone A	Aromatic Heterocyclic	Indole
Paraherquamide	Aromatic Heterocyclic	Indolizines
Paspaline	Aromatic Heterocyclic	Indole
Paspalinine	Aromatic Heterocyclic	Indole
Preechinulin	Aromatic Heterocyclic	Indole
Sclerotiamide	Aromatic Heterocyclic	Indolizines
Speradine A	Aromatic Heterocyclic	Indole
Verruculogen	Aromatic Heterocyclic	Indole
Versicoloid A	Aromatic Heterocyclic	Indole
Versicoloid B	Aromatic Heterocyclic	Indole
2''-oxoasterriquinol D Me ether	Aromatic Heterocyclic	Pyrans
TetrathioAspirochlorinee	Aromatic Heterocyclic	Pyrans
3-methyl-6-methoxy-8hydroxy-3,4-dihydroisocoumarin	Aromatic Heterocyclic	Benzopyrans
6, 8-dihydroxy-3-methylisocoumarin	Aromatic Heterocyclic	Benzopyrans
6,8-dihydroxy-3,7-dimethylisocoumarin	Aromatic Heterocyclic	Benzopyrans
Aflatoxin	Aromatic Heterocyclic	Benzopyrans
Aflatoxin B	Aromatic Heterocyclic	Benzopyrans
Aflatoxin B1	Aromatic Heterocyclic	Benzopyrans
Aflatoxin B2b	Aromatic Heterocyclic	Benzopyrans
Aflatoxin B3	Aromatic Heterocyclic	Benzopyrans
Aflatoxin D1	Aromatic Heterocyclic	Benzopyrans
Aflatoxin G	Aromatic Heterocyclic	Benzopyrans
Aflatoxin G2a	Aromatic Heterocyclic	Benzopyrans
Aflatoxin M1	Aromatic Heterocyclic	Benzopyrans
Aflatoxin M2	Aromatic Heterocyclic	Benzopyrans
Aflatoxin P1	Aromatic Heterocyclic	Benzopyrans
Aflatoxin Q1	Aromatic Heterocyclic	Benzopyrans
Aflatoxin-B2a	Aromatic Heterocyclic	Benzopyrans
Asperbiphenyl	Aromatic Heterocyclic	Benzopyrans
Aurasperone A	Aromatic Heterocyclic	Benzopyrans
Aurasperone B	Aromatic Heterocyclic	Benzopyrans
Aurasperone D	Aromatic Heterocyclic	Benzopyrans
Austdiol	Aromatic Heterocyclic	Benzopyrans
Austocystin	Aromatic Heterocyclic	Benzopyrans
Austocystin A	Aromatic Heterocyclic	Benzopyrans
Canescin	Aromatic Heterocyclic	Benzopyrans
Chlorflavonin	Aromatic Heterocyclic	Benzopyrans
Dihydrocitrinone	Aromatic Heterocyclic	Benzopyrans
Dihydrogeodin	Aromatic Heterocyclic	Benzopyrans
Flavipesin A	Aromatic Heterocyclic	Benzopyrans
Flavipesin B	Aromatic Heterocyclic	Benzopyrans
Isokotanin A	Aromatic Heterocyclic	Benzopyrans
Isokotanin B	Aromatic Heterocyclic	Benzopyrans

Isokotanin C	Aromatic Heterocyclic	Benzopyrans
Kotanin	Aromatic Heterocyclic	Benzopyrans
Leporin A	Aromatic Heterocyclic	Benzopyrans
Leporizine A	Aromatic Heterocyclic	Benzopyrans
Leporizine B	Aromatic Heterocyclic	Benzopyrans
Leporizine C	Aromatic Heterocyclic	Benzopyrans
Mellein	Aromatic Heterocyclic	Benzopyrans
Parasiticol	Aromatic Heterocyclic	Benzopyrans
Peniciphenol	Aromatic Heterocyclic	Benzopyrans
Pergillin	Aromatic Heterocyclic	Benzopyrans
Phenprocoumon	Aromatic Heterocyclic	Benzopyrans
Pseudodeflectusin	Aromatic Heterocyclic	Benzopyrans
Reticulol	Aromatic Heterocyclic	Benzopyrans
Rubrofusarin	Aromatic Heterocyclic	Benzopyrans
Sclerin	Aromatic Heterocyclic	Benzopyrans
Sclerotiotide F	Aromatic Heterocyclic	Benzopyrans
Silybin A	Aromatic Heterocyclic	Benzopyrans
TMC-256A1	Aromatic Heterocyclic	Benzopyrans
TMC-256C1	Aromatic Heterocyclic	Benzopyrans
TMC-2A	Aromatic Heterocyclic	Benzopyrans
TMC-2A	Aromatic Heterocyclic	Benzopyrans
TMC-2B	Aromatic Heterocyclic	Benzopyrans
TMC-2B	Aromatic Heterocyclic	Benzopyrans
TMC-2C	Aromatic Heterocyclic	Benzopyrans
TMC-2C	Aromatic Heterocyclic	Benzopyrans
Warfarin	Aromatic Heterocyclic	Benzopyrans
Silybin B	Aromatic Heterocyclic	Benzopyran
**12β**-hydroxy-**13α**-methoxyverruculogen TR-2	Aromatic Heterocyclic	Alkaloids
13-desoxypaxilline	Aromatic Heterocyclic	Alkaloids
14-epi-14-hydroxy-10,23-dihydro-24,25-dehydroaflavinine	Aromatic Heterocyclic	Alkaloids
23-Dihydro-24,25-dehydro-21-oxo-aflavinine	Aromatic Heterocyclic	Alkaloids
23-Dihydro-24,25-dehydroaflavinin	Aromatic Heterocyclic	Alkaloids
5,7-Dihydroxy-6-methylphthalide	Aromatic Heterocyclic	Benzofuran
Dithiosilvatin	Aromatic Heterocyclic	Alkaloids
Festuclavine	Aromatic Heterocyclic	Alkaloids
Flavasperone	Aromatic Heterocyclic	Alkaloids
Fumimycin	Aromatic Heterocyclic	Benzofuran
Geodin	Aromatic Heterocyclic	Benzofuran
Griseofulvin	Aromatic Heterocyclic	Benzofuran
Karnatakafuran A	Aromatic Heterocyclic	Benzofuran
Karnatakafuran B	Aromatic Heterocyclic	Benzofuran
Neoxaline	Aromatic Heterocyclic	Alkaloids
Nidulin	Aromatic Heterocyclic	Dibenzoxepins
Sequoiatone	Aromatic Heterocyclic	Heterocyclic compounds
Sequoiatone B	Aromatic Heterocyclic	Heterocyclic compounds
Sequoiatone A	Aromatic Heterocyclic	Heterocyclic compounds
3-hydroxyfumiquinazoline A	Aromatic Heterocyclic	Alkaloids
Kipukasin H	Aromatic Heterocyclic	Pyrimidines
Kipukasin I	Aromatic Heterocyclic	Pyrimidines

Kojistatin	Aromatic Heterocyclic	Pyrimidines
Apernigrin A	Aromatic Heterocyclic	Pyridine
Apernigrin B	Aromatic Heterocyclic	Pyridine
Aspertoxin	Aromatic Heterocyclic	Xanthenes
Asperdurin	Aromatic Heterocyclic	Benzofurans
Asperfuranone	Aromatic Heterocyclic	Benzofurans
Cyclopaldic acid	Aromatic Heterocyclic	Benzofurans
Cyclopenol	Aromatic Heterocyclic	Benzofurans
Cycloleucomelon	Aromatic Heterocyclic	Terpenyls
Asperxanthone	Aromatic Heterocyclic	Xanthone
Austradixanthone	Aromatic Heterocyclic	Xanthone
Avenaciolide	Aromatic Heterocyclic	Xanthone
cycloisoemericellin	Aromatic Heterocyclic	Xanthone
Emerixanthone B	Aromatic Heterocyclic	Xanthone
Emerixanthone C	Aromatic Heterocyclic	Xanthone
Emerixanthone A	Aromatic Heterocyclic	Xanthone
Emerixanthone D	Aromatic Heterocyclic	Xanthone
Secalonic acid B	Aromatic Heterocyclic	Xanthone
Secalonic Acid D	Aromatic Heterocyclic	Xanthone
Sydowinin A	Aromatic Heterocyclic	Xanthone
Sydowinin B	Aromatic Heterocyclic	Xanthone
Sydowinol	Aromatic Heterocyclic	Xanthone
Variecoxanthone A	Aromatic Heterocyclic	Xanthone
Variecoxanthone B	Aromatic Heterocyclic	Xanthone
Variecoxanthone C	Aromatic Heterocyclic	Xanthone
3-O-methylsterigmatocystin	Aromatic Heterocyclic	Xanthanes
5-methoxysterigmatocystin	Aromatic Heterocyclic	Xanthenes
Dihydrosterigmatocystin	Aromatic Heterocyclic	Xanthenes
O-Methylsterigmatocystin	Aromatic Heterocyclic	Xanthenes
Secalonic Acid F	Aromatic Heterocyclic	Xanthenes
Asperrubrol	Aromatic Heterocyclic	Phenylpolyenes
Viridicatumtoxin	Aromatic Heterocyclic Amide	Carboxamide
4'-OMe-asperphenamate	Aromatic Heterocyclic Amino acid	Phenylalanine
Cotteslosin A	Aromatic Heterocyclic Amino acid	Tryosine
Cotteslosin B	Aromatic Heterocyclic Amino acid	Tryosine
Cyclo(D-N-methyl-Leu-L-Trp)	Aromatic Heterocyclic Amino acid	Tryptophan
Mulundocandin	Aromatic Heterocyclic Amino acid	Cyclic Peptides
Psychrophilin E	Aromatic Heterocyclic Amino acid	Cyclic Peptides
Similanamide	Aromatic Heterocyclic Amino acid	Cyclic Peptides
Asperparaline A	Aromatic Heterocyclic Amino acid	Phenylalanine
Asperparaline B	Aromatic Heterocyclic Amino acid	Phenylalanine
Asperparalines C	Aromatic Heterocyclic Amino acid	Phenylalanine
Asperphenamate	Aromatic Heterocyclic Amino acid	Phenylalanine
Asperpyrone B	Aromatic Heterocyclic Amino acid	Phenylalanine

Asperpyrone C	Aromatic Heterocyclic Amino acid	Phenylalanine
Asperpyrone A	Aromatic Heterocyclic Amino acid	Phenylalanine
Cristatin A	Aromatic Heterocyclic Amino acid	Tryptophan
Cristatin A	Aromatic Heterocyclic Amino acid	Tryptophan
Ditryptophenaline	Aromatic Heterocyclic Amino acid	Phenylalanine
Lovastatin	Aromatic Heterocyclic Amino acid	Phenylalanine
Ochratoxin	Aromatic Heterocyclic Amino acid	Phenylalanine
Ochratoxin A	Aromatic Heterocyclic Amino acid	Phenylalanine
Ochratoxin B	Aromatic Heterocyclic Amino acid	Phenylalanine
Ochratoxin C	Aromatic Heterocyclic Amino acid	Phenylalanine
Ochrindole A	Aromatic Heterocyclic Amino acid	Phenylalanine
Ochrindole B	Aromatic Heterocyclic Amino acid	Phenylalanine
Ochrindole C	Aromatic Heterocyclic Amino acid	Phenylalanine
Ochrindole D	Aromatic Heterocyclic Amino acid	Phenylalanine
Malformin	Aromatic Heterocyclic Amino acid	Cyclic Peptides
Malformin A	Aromatic Heterocyclic Amino acid	Cyclic Peptides
Malformin A1	Aromatic Heterocyclic Amino acid	Cyclic Peptides
Malformin A2	Aromatic Heterocyclic Amino acid	Cyclic Peptides
Malformin B1a	Aromatic Heterocyclic Amino acid	Cyclic Peptides
Malformin B2	Aromatic Heterocyclic Amino acid	Cyclic Peptides
Malformin B3	Aromatic Heterocyclic Amino acid	Cyclic Peptides
Malformin C	Aromatic Heterocyclic Amino acid	Cyclic Peptides
Malformin E	Aromatic Heterocyclic Amino acid	Cyclic Peptides
Alanyltryptophan anhydride	Aromatic Heterocyclic Anhydrides	Anhydrides
Cycloechinulin	Aromatic Heterocyclic Diamene	Piperazines
Emestrin	Aromatic Heterocyclic Diamene	Piperazines
Emethacin B	Aromatic Heterocyclic Diamene	Piperazines
Ent-cycloechinulin	Aromatic Heterocyclic Diamene	Piperazines
Neoechinulin A	Aromatic Heterocyclic Diamene	Piperazines
Neoechinulin B	Aromatic Heterocyclic Diamene	Piperazines
Novoamauromine	Aromatic Heterocyclic Diamene	Piperazines
Phenylahistin	Aromatic Heterocyclic Diamene	Piperazines
Aspergillic acid	Aromatic Heterocyclic Diamene	Pyrazines
5-N-acetyl-15b-didehydroardeemin	Aromatic Heterocyclic Diazene	Pyrimidinones
5-N-acetyl-**15bβ**-hydroxyardeemin	Aromatic Heterocyclic Diazene	Pyrimidinones
5-N-acetyl-**16α**-hydroxyardeemin	Aromatic Heterocyclic Diazene	Pyrimidinones

5-N-acetylardeemin	Aromatic Heterocyclic Diazene	Pyrimidinones
5-O-methylsclerone	Aromatic Heterocyclic Diazene	Pyrimidinones
6-Deoxyversicolorin A	Aromatic Heterocyclic Diketone	Quinones
8-O-methylversicolorin A	Aromatic Heterocyclic Diketone	Quinones
8-O-methylversicolorin B	Aromatic Heterocyclic Diketone	Quinones
Variecolorquinone A	Aromatic Heterocyclic Diketone	Quinones
Variecolorquinone B	Aromatic Heterocyclic Diketone	Quinones
Aspernomine	Aromatic Heterocyclic Hydrocarbons	Diterpene
Asperolide A	Aromatic Heterocyclic Hydrocarbons	Diterpene
Asperolide B	Aromatic Heterocyclic Hydrocarbons	Diterpene
Asperolide D	Aromatic Heterocyclic Hydrocarbons	Diterpene
Asperolide E	Aromatic Heterocyclic Hydrocarbons	Diterpene
Aspewentin A	Aromatic Heterocyclic Hydrocarbons	Diterpene
Aspewentin B	Aromatic Heterocyclic Hydrocarbons	Diterpene
Aspewentin C	Aromatic Heterocyclic Hydrocarbons	Diterpene
Aspewentin D	Aromatic Heterocyclic Hydrocarbons	Diterpene
Aspewentin E	Aromatic Heterocyclic Hydrocarbons	Diterpene
Aspewentin F	Aromatic Heterocyclic Hydrocarbons	Diterpene
Aspewentin G	Aromatic Heterocyclic Hydrocarbons	Diterpene
Aspewentin H	Aromatic Heterocyclic Hydrocarbons	Diterpene
Aspirochlorine	Aromatic Heterocyclic Hydrocarbons	Hydrocarbons
Astellolide A	Aromatic Heterocyclic Hydrocarbons	Sesquiterpenes
Astellolide B	Aromatic Heterocyclic Hydrocarbons	Sesquiterpenes
Austrosene	Aromatic Heterocyclic Hydrocarbons	Sesquiterpenes
Aversin	Aromatic Heterocyclic Hydrocarbons	Sesquiterpenes
Drimane sesquiterpenoid	Aromatic Heterocyclic Hydrocarbons	Sesquiterpenes
Drimane	Aromatic Heterocyclic Hydrocarbons	Sesquiterpenes
Echinuline	Aromatic Heterocyclic Hydrocarbons	Terpene
Hydroxysydonic acid	Aromatic Heterocyclic Hydrocarbons	Sesquiterpene
Hydroxyyanuthone A	Aromatic Heterocyclic Hydrocarbons	Terpenes
Hydroxyyanuthone C	Aromatic Heterocyclic Hydrocarbons	Terpenes
Nominine	Aromatic Heterocyclic Hydrocarbons	Diterpene
Penitrem	Aromatic Heterocyclic Hydrocarbons	Diterpene
Carboxydiphenylbutenoic anhydride	Aromatic Heterocyclic Hydroxy pyridine	Pyridones
Carneamide A	Aromatic Heterocyclic Hydroxy	Pyridones

	pyridine	
Carneamide B	Aromatic Heterocyclic Hydroxy pyridine	Pyridones
Carneamide C	Aromatic Heterocyclic Hydroxy pyridine	Pyridones
Oxaline	Aromatic Heterocyclic Imidazole	Imidazole
3,6-dihydroxytoluquinone	Aromatic Heterocyclic Ketone	Quinones
9-chloro-8-hydroxy-8,9-deoxyaspyrone	Aromatic Heterocyclic Ketone	Pyrones
Astechrome	Aromatic Heterocyclic Ketone	Pyrones
Campyrone A	Aromatic Heterocyclic Ketone	Pyrones
Campyrone C	Aromatic Heterocyclic Ketone	Pyrones
Carbonarone A	Aromatic Heterocyclic Ketone	Pyrones
Carbonarone B	Aromatic Heterocyclic Ketone	Pyrones
Chevalone B	Aromatic Heterocyclic Ketone	Pyrans
Chevalone C	Aromatic Heterocyclic Ketone	Pyrans
Chevalone E	Aromatic Heterocyclic Ketone	Pyrans
Dechloronidulin	Aromatic Heterocyclic Ketone	Pyrones
Deoxyaverufinone	Aromatic Heterocyclic Ketone	Pyran
Foncesinone A	Aromatic Heterocyclic Ketone	Pyrones
Fonsecinone B	Aromatic Heterocyclic Ketone	Pyrones
Fonsecinone C	Aromatic Heterocyclic Ketone	Pyrones
Fonsecinone D	Aromatic Heterocyclic Ketone	Pyrones
Naphtho-**γ**-pyrone	Aromatic Heterocyclic Ketone	Pyrones
Nigerapyrone A	Aromatic Heterocyclic Ketone	Pyrones
Nigerapyrone B	Aromatic Heterocyclic Ketone	Pyrones
Nigerapyrone C	Aromatic Heterocyclic Ketone	Pyrones
Nigerapyrone D	Aromatic Heterocyclic Ketone	Pyrones
Nigerapyrone E	Aromatic Heterocyclic Ketone	Pyrones
Nigerapyrone F	Aromatic Heterocyclic Ketone	Pyrones
Nigerapyrone G	Aromatic Heterocyclic Ketone	Pyrones
Nigerapyrone H	Aromatic Heterocyclic Ketone	Pyrones
Parasperone	Aromatic Heterocyclic Ketone	Pyrone
Pyranonigrin A	Aromatic Heterocyclic Ketone	Pyrones
Pyrophen	Aromatic Heterocyclic Ketone	Pyrones
Similanpyrone A	Aromatic Heterocyclic Ketone	Pyrones
Similanpyrone B	Aromatic Heterocyclic Ketone	Pyrones
Similanpyrone C	Aromatic Heterocyclic Ketone	Pyrones
Sydowic acid	Aromatic Heterocyclic Ketone	Pyran
11-O-methylpseurotin A	Aromatic Heterocyclic Ketone	Pyrrolidinones
Pseurotin	Aromatic Heterocyclic Ketone	Pyrrolidinones
4,7-dimethoxy-5-methylcoumarin	Aromatic Heterocyclic Ketone	Pyrans
4-hydroxymethyl-5-hydroxy-2H-pyran-2-one	Aromatic Heterocyclic Ketone	Pyrans
4-hydroxymethyl-5-hydroxy-2H-pyran-2-one	Aromatic Heterocyclic Ketone	Pyrans
5,6-Dimethoxydihydrosterigmatocystin	Aromatic Heterocyclic Ketone	Pyrans
Cladosporin	Aromatic Heterocyclic Ketone	Pyrans
Coniochaetone A	Aromatic Heterocyclic Ketone	Pyrans
Meroterpenoid	Aromatic Heterocyclic Ketone	Pyrans
Patulin	Aromatic Heterocyclic Ketone	Pyrans
Gregatin	Aromatic Heterocyclic Ketone	Furans
Ascladiol	Aromatic Heterocyclic Ketone	Furans

Asnipyrone A	Aromatic Heterocyclic Ketone	Furans
Asnipyrone B	Aromatic Heterocyclic Ketone	Furans
Aspulvinone	Aromatic Heterocyclic Ketone	Furans
Aspulvinone D	Aromatic Heterocyclic Ketone	Furans
Cyclogregatin	Aromatic Heterocyclic Ketone	Furans
Gregatin A	Aromatic Heterocyclic Ketone	Furans
Gregatin B	Aromatic Heterocyclic Ketone	Furans
Gregatin D	Aromatic Heterocyclic Ketone	Furans
Tensidol A	Aromatic Heterocyclic Ketone	Furans
Tensidol B	Aromatic Heterocyclic Ketone	Furans
Tetronic acid	Aromatic Heterocyclic Ketone	Furans
5,6-dihydro-5(S)-acetoxy-6(S)-(1,2-trans propenyl)-2H-pyran-2-one	Aromatic Heterocyclic Ketone	Pyrans
5-hydroxy-8-methyl-2H, 6H-pyrano[3,4-g]chromen-2,6-dione	Aromatic Heterocyclic Ketone	Pyrans
O-ethylparvulenone	Aromatic Heterocyclic Ketone	Ketone
4-(N-methyl-N-phenylamino) butan-2-one	Aromatic Heterocyclic Ketone	Ketones
Amodin A	Aromatic Heterocyclic Ketone	Ketone
Amodin B	Aromatic Heterocyclic Ketone	Ketone
Asperasone	Aromatic Heterocyclic Ketone	Diketone
Aspersitin	Aromatic Heterocyclic Ketone	Ketone
Aspiketolactonol	Aromatic Heterocyclic Ketone	Polyketones
Bis-indoyl benzenoid	Aromatic Heterocyclic Ketone	Ketones
Desmethylkotanin	Aromatic Heterocyclic Ketone	Diketone
Erythroglaucin	Aromatic Heterocyclic Ketone	Quinone
Funalenone	Aromatic Heterocyclic Ketone	Ketones
Viomellin	Aromatic Heterocyclic Ketone	Trione
Deoxypodophyllotoxin	Aromatic Heterocyclic Polysaccrides	Lignans
Desacetylaustin	Aromatic Heterocyclic Polysaccrides	Lignans
Sterigmatocystin	Aromatic Heterocyclic Purines	Xanthines
Aflatoxin G1	Aromatic Heterocyclic Purines	Benzopyrans
Aflatoxin G2	Aromatic Heterocyclic Purines	Benzopyrans
Citreoisocoumarin	Aromatic Heterocyclic Purines	Benzopyrans
Citreoviridin	Aromatic Heterocyclic Purines	Aurovertins
Citrinin	Aromatic Heterocyclic Purines	Benzopyrans
Fulvic acid	Aromatic Heterocyclic Purines	Benzopyrans
Pestalamide C	Aromatic Heterocyclic Purines	Pyranone
2-hydroxy-3-methyl-1,4-benzoquinone	Aromatic Heterocyclic Quinone	Benzo quinone
6,8,1'-tri-O-methyl averantin	Aromatic Heterocyclic Quinone	Anthraquinones
6,8-di-O-methylaverufin	Aromatic Heterocyclic Quinone	Anthraquinones
8-O-methylaverythin	Aromatic Heterocyclic Quinone	Anthraquinones
Aspergiodiquinone	Aromatic Heterocyclic Quinone	Napthoquinones
Aspergluacide	Aromatic Heterocyclic Quinone	Napthoquinones
Asperthecin	Aromatic Heterocyclic Quinone	Anthraquinones
Averufin	Aromatic Heterocyclic Quinone	Anthraquinones
Averythrin	Aromatic Heterocyclic Quinone	Anthraquinones
Chrysophanol	Aromatic Heterocyclic Quinone	Anthraquinones
Cichorine	Aromatic Heterocyclic Quinone	Anthraquinones
Emodin	Aromatic Heterocyclic Quinone	Anthraquinones
Pachybasin	Aromatic Heterocyclic Quinone	Anthraquinones
Physcion	Aromatic Heterocyclic Quinone	Anthraquinones

Questin	Aromatic Heterocyclic Quinone	Anthraquinones
Versicolorin	Aromatic Heterocyclic Quinone	Anthraquinones
Violaceoid A	Aromatic Heterocyclic Quinone	Hydroquinones
Violaceoid B	Aromatic Heterocyclic Quinone	Hydroquinones
Violaceoid C	Aromatic Heterocyclic Quinone	Hydroquinones
Violaceoid D	Aromatic Heterocyclic Quinone	Hydroquinones
Violaceoid E	Aromatic Heterocyclic Quinone	Hydroquinones
Viridicatol	Aromatic Heterocyclic Quinone	Hydroxyquinones
4-methoxybenzyl 7-phenylacetamido-3-vinyl-3-cephem-4-carboxylate	Aromatic Heterocyclic Vitamin D	Calcitrol
**5α,8α**-epidioxyergosta-6,22-dien-**3β**-ol	Aromatic Heterocyclic Vitamin D	Ergosterol
Isocyathisterol	Aromatic Heterocyclic Vitamin D	Ergosterol
Rosellichalasin	Aromatic Heterocyclic Vitamin D	Ergo sterol
Violaceol I	Aromatic Homocyclic	Ethers
Violaceol II	Aromatic Homocyclic	Ethers
Violaceol II	Aromatic Homocyclic	Ethers
Iizukine A	Aromatic hydrocarbon Heterocyclic	Diphenyl
Iizukine B	Aromatic hydrocarbon Heterocyclic	Diphenyl
2-methoxy-6-(3,4-dihydroxyhepta-1,5-dienyl)benzyl alcohol	Aromatic hydrocarbon Heterocyclic	Benzyl derivatives
3-Hydroxyterphenyllin	Aromatic hydrocarbon Heterocyclic	Phenylbenzene
4''-dehydro-3-hydroxyterphenyllin	Aromatic hydrocarbon Heterocyclic	Phenylbenzene
Aspergentisyl A	Aromatic hydrocarbon Heterocyclic	Benzyl derivatives
Aspergentisyl B	Aromatic hydrocarbon Heterocyclic	Benzyl derivatives
Aspergiketal	Aromatic hydrocarbon Heterocyclic	Benzyl derivatives
Aspergillazine A	Aromatic hydrocarbon Heterocyclic	Benzyl derivatives
Aspergillazine B	Aromatic hydrocarbon Heterocyclic	Benzyl derivatives
Aspergillazine C	Aromatic hydrocarbon Heterocyclic	Benzyl derivatives
Aspergillazine D	Aromatic hydrocarbon Heterocyclic	Benzyl derivatives
Aspergillazine E	Aromatic hydrocarbon Heterocyclic	Benzyl derivatives
Candidusin A	Aromatic hydrocarbon Heterocyclic	Phenylbenzene
Candidusin C	Aromatic hydrocarbon Heterocyclic	Phenylbenzene
Candidusin B	Aromatic hydrocarbon Heterocyclic	Phenylbenzene
Carbonarin A	Aromatic hydrocarbon Heterocyclic	Phenylbenzene
Carbonarin B	Aromatic hydrocarbon Heterocyclic	Phenylbenzene
Carbonarin C	Aromatic hydrocarbon Heterocyclic	Phenylbenzene
Carbonarin D	Aromatic hydrocarbon Heterocyclic	Phenylbenzene
Carbonarin E	Aromatic hydrocarbon Heterocyclic	Phenylbenzene

Carbonarin F	Aromatic hydrocarbon Heterocyclic	Phenylbenzene
Carbonarin G	Aromatic hydrocarbon Heterocyclic	Phenylbenzene
Carbonarin H	Aromatic hydrocarbon Heterocyclic	Phenylbenzene
Carbonarin I	Aromatic hydrocarbon Heterocyclic	Phenylbenzene
Carbonarin J	Aromatic hydrocarbon Heterocyclic	Phenylbenzene
Prenylcandidusin A	Aromatic hydrocarbon Heterocyclic	Phenylbenzene
Prenylcandidusin B	Aromatic hydrocarbon Heterocyclic	Phenylbenzene
Prenylcandidusin C	Aromatic hydrocarbon Heterocyclic	Phenylbenzene
Prenylterphenyllin A	Aromatic hydrocarbon Heterocyclic	Phenylbenzene
Prenylterphenyllin B	Aromatic hydrocarbon Heterocyclic	Phenylbenzene
Prenylterphenyllin C	Aromatic hydrocarbon Heterocyclic	Phenylbenzene
p-hydroxybenzaldehyde	Aromatic aldehyde Homocyclic	Benzaldehydes
Prenylated benzaldehyde	Aromatic aldehyde Homocyclic	Benzaldehydes
(R)-2-(hydroxymethyl)-3-(2-hydroxypropyl)pheno,	Benzene derivatives Aromatic Heterocyclic	Phenols
2-O-[14C]Methylorsellinic acid	Benzene derivatives Aromatic Heterocyclic	Phenols
3-methylorsellinate	Benzene derivatives Aromatic Heterocyclic	Phenols
Arenarin A	Benzene derivatives Aromatic Heterocyclic	Phenols
Arenarin B	Benzene derivatives Aromatic Heterocyclic	Phenols
Arenarin C	Benzene derivatives Aromatic Heterocyclic	Phenols
Arugosin	Benzene derivatives Aromatic Heterocyclic	Phenols
Arugosin C	Benzene derivatives Aromatic Heterocyclic	Phenols
Arugosin D	Benzene derivatives Aromatic Heterocyclic	Phenols
Arugosin E	Benzene derivatives Aromatic Heterocyclic	Phenols
Arugosin G	Benzene derivatives Aromatic Heterocyclic	Phenols
Arugosin H	Benzene derivatives Aromatic Heterocyclic	Phenols
Arugosin K	Benzene derivatives Aromatic Heterocyclic	Phenols
Arugosin A	Benzene derivatives Aromatic Heterocyclic	Phenols
Arugosin B	Benzene derivatives Aromatic Heterocyclic	Phenols
Dehydrocurvularin	Benzene derivatives Aromatic Heterocyclic	Phenols
Diorcinol	Benzene derivatives Aromatic Heterocyclic	Phenols
O-Methylasparvenone	Benzene derivatives Aromatic Heterocyclic	Napthols
Oxoasterriquinol D	Benzene derivatives Aromatic Heterocyclic	Benzoid
Mevmevinolin	Benzene derivatives Aromatic	Phenol

	Heterocyclic	
Carnequinazoline A	Benzopyrimidine Aromatic Heterocyclic	Quinazoline
Carnequinazoline B	Benzopyrimidine Aromatic Heterocyclic	Quinazoline
Carnequinazoline C	Benzopyrimidine Aromatic Heterocyclic	Quinazoline
Chaetoquadrin F	Benzopyrimidine Aromatic Heterocyclic	Quinazoline
Chrysogine	Benzopyrimidine Aromatic Heterocyclic	Quinazoline
Cottoquinazoline A	Benzopyrimidine Aromatic Heterocyclic	Quinazoline
Fumiquinazoline A	Benzopyrimidine Aromatic Heterocyclic	Quinazoline
Fumiquinazoline C	Benzopyrimidine Aromatic Heterocyclic	Quinazoline
Quinazolinone	Benzopyrimidine Aromatic Heterocyclic	Quinazoline
Regulin	Benzopyrimidine Aromatic Heterocyclic	Quinazoline
3-Nitropropionic acid	Carboxylic acid Acyclic	Propionates
E-2-decenedioic acid	Carboxylic acid Acyclic	Fumaric acid
Ethanedioc acid	Carboxylic acid Acyclic	Oxolates
2-Methylene-3hexylbutanedioic acid	Carboxylic acid Acyclic	Carboxylic acid
3-hydroxy-5-methoxy-3-(methoxycarbonyl)-5-oxopentanoic acid	Carboxylic acid Acyclic	Tricarboxylic acids
Aculinic acid	Carboxylic acid Acyclic	Fatty acids
Asperic acid	Carboxylic acid Acyclic	Fatty acids
Velutinin	Carboxylic acid Acyclic	Carboxylic acid
Erdin	Carboxylic acid Acyclic	Ester
Citric acid	Carboxylic acid Acyclic	Tricarboxylic acids
Fumaric acid	Carboxylic acid Acyclic	Dicarboxylic acid
Fumonisin	Carboxylic acid Acyclic	Carboxylic acid
Fumonisin B4	Carboxylic acid Acyclic	Carboxylic acid
Fumonisin B2	Carboxylic acid Acyclic	Carboxylic acid
Hexanoic acid	Carboxylic acid Acyclic	Caproates
Hexylitaconic acid	Carboxylic acid Acyclic	Succinates
Itaconic acid	Carboxylic acid Acyclic	Succinates
Oxalic acid	Carboxylic acid Acyclic	Dicarboxylic acid
Phthioic acid	Carboxylic acid Acyclic	Fatty acids
Succinic acid	Carboxylic acid Acyclic	Succinates
Tensyuic acid A	Carboxylic acid Acyclic	Dicarboxylic acid
Tensyuic acid B	Carboxylic acid Acyclic	Dicarboxylic acid
Tensyuic acid C	Carboxylic acid Acyclic	Dicarboxylic acid
Tensyuic acid D	Carboxylic acid Acyclic	Dicarboxylic acid
Tensyuic acid E	Carboxylic acid Acyclic	Dicarboxylic acid
Tensyuic acid F	Carboxylic acid Acyclic	Dicarboxylic acid
2-(3-chloro-4-methyl-**γ**-resorcyloyl)-5-hydroxy-m-anisic acid methyl ester	Carboxylic acid Alicyclic Heterocyclic	Methyl ester
2-Carboxymethyl-3-hexylmaleic acid anhydride	Carboxylic acid Alicyclic Heterocyclic	Acid anhydride
3-Furanacetic acid	Carboxylic acid Alicyclic Heterocyclic	Acetates
**Monacolin L β**-hydroxy acid	Carboxylic acid Alicyclic Heterocyclic	Carboxylic acid
Ergosterol	Carboxylic acid Alicyclic	Cholestans

	Heterocyclic	
(7E,7'E)-5,5'-diferulic acid	Carboxylic acid Aromatic Heterocyclic	Cinnamates
Asperugin	Carboxylic acid Aromatic Heterocyclic	Aldehydes
Asperversin A	Carboxylic acid Aromatic Heterocyclic	Carbamates
Austalide O	Carboxylic acid Aromatic Heterocyclic	Esters
Austalide P	Carboxylic acid Aromatic Heterocyclic	Esters
Austalide Q	Carboxylic acid Aromatic Heterocyclic	Esters
Austalide R	Carboxylic acid Aromatic Heterocyclic	Esters
Austalide A	Carboxylic acid Aromatic Heterocyclic	Esters
Austalide B	Carboxylic acid Aromatic Heterocyclic	Esters
Austalide C	Carboxylic acid Aromatic Heterocyclic	Esters
Austalide D	Carboxylic acid Aromatic Heterocyclic	Esters
Austalide E	Carboxylic acid Aromatic Heterocyclic	Esters
Austalide F	Carboxylic acid Aromatic Heterocyclic	Esters
Austalide G	Carboxylic acid Aromatic Heterocyclic	Esters
Austalide H	Carboxylic acid Aromatic Heterocyclic	Esters
Austalide I	Carboxylic acid Aromatic Heterocyclic	Esters
Austalide J	Carboxylic acid Aromatic Heterocyclic	Esters
Austalide K	Carboxylic acid Aromatic Heterocyclic	Esters
Austalide L	Carboxylic acid Aromatic Heterocyclic	Esters
Austalide M	Carboxylic acid Aromatic Heterocyclic	Esters
Austalide N	Carboxylic acid Aromatic Heterocyclic	Esters
Isodihydroauroglaucin 2	Carboxylic acid Aromatic Heterocyclic	Benzoates
Isotetrahydroauroglaucin	Carboxylic acid Aromatic Heterocyclic	Benzoates
Monomethylsulochrin	Carboxylic acid Aromatic Heterocyclic	Benzoates
Auroglaucine	Carboxylic acid Aromatic Heterocyclic	Benzoates
Dihydroauroglaucin	Carboxylic acid Aromatic Heterocyclic	Benzoates
Flavoglaucin	Carboxylic acid Aromatic Heterocyclic	Benzoates
Isodihydroauroglaucin 1	Carboxylic acid Aromatic Heterocyclic	Benzoates
Isosulochrin	Carboxylic acid Aromatic Heterocyclic	Benzoates
Isosulochrin dehydrate	Carboxylic acid Aromatic Heterocyclic	Benzoates
Sulochrin	Carboxylic acid Aromatic Heterocyclic	Benzoates

Ustic acid	Carboxylic acid Aromatic Heterocyclic	Benzoic acid
Ustilaginoidin C	Carboxylic acid Aromatic Heterocyclic	Pyrans
Phenyl dioxolanone	Carboxylic acid Aromatic Heterocyclic	Benzoic acid
Penicillic acid	Cyclic Ester Acyclic	Lactones
(*S*)-2-**(2′**-hydroxyethyl)-4-methyl-**γ**-butyrolactone	Cyclic ester Alicyclic Heterocyclic	Lactone
6-O-methylaverufin	Cyclic ester Alicyclic Heterocyclic	Lactones
8-chloro-9-hydroxy-8,9-deoxyasperlactone	Cyclic ester Alicyclic Heterocyclic	Lactones
Apiaspinonediol	Cyclic ester Alicyclic Heterocyclic	Lactone
Aspilactonol A	Cyclic ester Alicyclic Heterocyclic	Lactone
Aspilactonol B	Cyclic ester Alicyclic Heterocyclic	Lactone
Aspilactonol C	Cyclic ester Alicyclic Heterocyclic	Lactone
Aspilactonol D	Cyclic ester Alicyclic Heterocyclic	Lactone
Aspilactonol E	Cyclic ester Alicyclic Heterocyclic	Lactone
Aspilactonol F	Cyclic ester Alicyclic Heterocyclic	Lactone
Butenolide	Cyclic ester Alicyclic Heterocyclic	Lactone
Butyrolactone	Cyclic ester Alicyclic Heterocyclic	Lactones
Butyrolactone I	Cyclic ester Alicyclic Heterocyclic	Lactones
Butyrolactone II	Cyclic ester Alicyclic Heterocyclic	Lactones
Butyrolactone III	Cyclic ester Alicyclic Heterocyclic	Lactones
Butyrolactone III 1,2-dichloroethane	Cyclic ester Alicyclic Heterocyclic	Lactones
Butyrolactone IV	Cyclic ester Alicyclic Heterocyclic	Lactones
Canadensolide	Cyclic ester Alicyclic Heterocyclic	Lactones
dihydropenicillic acid	Cyclic ester Alicyclic Heterocyclic	Lactones
Folipastatin	Cyclic ester Alicyclic Heterocyclic	Lactones
Monacolin L lactone	Cyclic ester Alicyclic Heterocyclic	Lactones
Monorden A	Cyclic ester Alicyclic Heterocyclic	Lactones
Monorden C	Cyclic ester Alicyclic Heterocyclic	Lactones
Monorden D	Cyclic ester Alicyclic Heterocyclic	Lactones
Monorden E	Cyclic ester Alicyclic Heterocyclic	Lactones
Penicillin	Cyclic ester Alicyclic Heterocyclic	Lactones
Wentilactone A	Cyclic ester Alicyclic Heterocyclic	Lactones
Wentilactone A	Cyclic ester Alicyclic Heterocyclic	Lactones

Wentilactone B	Cyclic ester Alicyclic Heterocyclic	Lactones
Xanthoherquein	Cyclic ester Alicyclic Heterocyclic	Lactones
Antafumicin A	Cyclic ester Aromatic Heterocyclic	Lactones
Antafumicin B	Cyclic ester Aromatic Heterocyclic	Lactones
Antibiotic Y	Cyclic ester Aromatic Heterocyclic	Lactones
Aryl C-glycoside	Cyclic ester Aromatic Heterocyclic	Lactone
Avertoxin A	Cyclic ester Aromatic Heterocyclic	Lactones
Avertoxin B	Cyclic ester Aromatic Heterocyclic	Lactones
Avertoxin C	Cyclic ester Aromatic Heterocyclic	Lactones
Avertoxin D	Cyclic ester Aromatic Heterocyclic	Lactones
Nidulol	Cyclic ester Aromatic Heterocyclic	Lactone
4R,5S-dihydroxy-3-methoxy-5-methylcyclohex-2-enone	Cyclic Hydrocarbons Alicyclic Heterocyclic	Cyclohexanes
Fumagillin	Cyclic Hydrocarbons Alicyclic Heterocyclic	Cyclohexanes
Parasitenone	Cyclic Hydrocarbons Alicyclic Heterocyclic	Cyclohexanes
Terrein	Cyclic Hydrocarbons Alicyclic Heterocyclic	Cyclopentanes
Benzophenone	Cyclic Hydrocarbons Aromatic Homocyclic	Hydrocarbons benzene
Aspergillicin A	Cyclic peptides Aromatic Heterocyclic	Cyclodepsipeptides
Aspergillicin B	Cyclic peptides Aromatic Heterocyclic	Cyclodepsipeptides
Aspergillicin C	Cyclic peptides Aromatic Heterocyclic	Cyclodepsipeptides
Aspergillicin D	Cyclic peptides Aromatic Heterocyclic	Cyclodepsipeptides
Aspergillicin E	Cyclic peptides Aromatic Heterocyclic	Cyclodepsipeptides
Aspergillin	Cyclic peptides Aromatic Heterocyclic	Cyclodepsipeptides
Aspergillin PZ	Cyclic peptides Aromatic Heterocyclic	Cyclodepsipeptides
Chloromethyresorcyloylhydroxyanisinate	Cyclic peptides Aromatic Heterocyclic	Methyl ester
Deflectin	Ester Aromatic Heterocyclic	Methylbenzoate
Deflectin A	Ester Aromatic Heterocyclic	Methylbenzoate
Deflectin B	Ester Aromatic Heterocyclic	Methylbenzoate
Ethyl atrarate	Ester Aromatic Heterocyclic	Ester
Neosartorin	Ester Aromatic Heterocyclic	Dicarboxylic acid
Sulochrin triacetate	Ester Aromatic Heterocyclic	Ester
Andibenin	Ether Alicyclic Heterocyclic	Methylethers
Andibenin A	Ether Alicyclic Heterocyclic	Methylethers
Andibenin B	Ether Alicyclic Heterocyclic	Methylethers
Andibenin C	Ether Alicyclic Heterocyclic	Methylethers
Asterriquinol D dimethyl ether	Ether Alicyclic Heterocyclic	Dimethyl ehther
Ditryptoleucine	Ether Alicyclic Heterocyclic	Ethers

Oxepin	Ether Alicyclic Heterocyclic	Ethers
Questin diacetate	Ether Alicyclic Heterocyclic	Methylethers
Asterric acid	Ether Aromatic Heterocyclic	Phenyl ethers
Ethericin A	Ether Aromatic Heterocyclic	Phenyl ethers
Ethericin B	Ether Aromatic Heterocyclic	Phenyl ethers
Ferulic acid	Ether Aromatic Heterocyclic	Phenyl ethers
Diphenyl ether	Ether Aromatic Homocyclic	Ethers
Zygosporin D	Ketone Acyclic	Diketone
7-ethyl-3,4-dihydro-4,6,8-trihydroxy-1(2H)-naphthalenone	Polycyclic aromatic Heterocyclic Hydrocarbon	Phenalenes
7-ethyl-3,4-dihydro-4,8-dihydroxy-6-methoxy-1(2H)-naphthalenone	Polycyclic aromatic Heterocyclic Hydrocarbon	Phenalenes
**8′**-O-Demethylnigerone	Polycyclic aromatic Heterocyclic Hydrocarbon	Phenalenes
Asparvenone	Polycyclic aromatic Heterocyclic Hydrocarbon	Phenalenes
Asperaculin A	Polycyclic aromatic Heterocyclic Hydrocarbon	Phenalenes
Atrovenetin	Polycyclic aromatic Heterocyclic Hydrocarbon	Phenalenes
Atrovenetin	Polycyclic aromatic Heterocyclic Hydrocarbon	Phenalenes
Auranthine	Polycyclic aromatic Heterocyclic Hydrocarbon	Phenalenes
Cis-4-Hydroxymellein	Polycyclic aromatic Heterocyclic Hydrocarbon	Phenalenes
Dianhydroaurasperone C	Polycyclic aromatic Heterocyclic Hydrocarbon	Phenalenes
Fonsecin monomethyl ether	Polycyclic aromatic Heterocyclic Hydrocarbon	Phenalenes
Isoaurasperone A	Polycyclic aromatic Heterocyclic Hydrocarbon	Phenalenes
Isoaurasperone A	Polycyclic aromatic Heterocyclic Hydrocarbon	Phenalenes
Mevastatin	Polycyclic aromatic Heterocyclic Hydrocarbon	Phenalenes
Mevinolin	Polycyclic aromatic Heterocyclic Hydrocarbon	Phenalenes
Mevinolinic acid	Polycyclic aromatic Heterocyclic Hydrocarbon	Phenalenes
Monacolin J	Polycyclic aromatic Heterocyclic Hydrocarbon	Phenalenes
Naphthalenone	Polycyclic aromatic Heterocyclic Hydrocarbon	Phenalenes
Naphthalic anhydride	Polycyclic aromatic Heterocyclic Hydrocarbon	Phenalenes
Naphthazarin epoxide	Polycyclic aromatic Heterocyclic Hydrocarbon	Phenalenes
Nigerasperone A	Polycyclic aromatic Heterocyclic Hydrocarbon	Phenalenes
Nigerasperone B	Polycyclic aromatic Heterocyclic Hydrocarbon	Phenalenes
Nigerasperone C	Polycyclic aromatic Heterocyclic Hydrocarbon	Phenalenes
Pravastatin	Polycyclic aromatic Heterocyclic Hydrocarbon	Phenalenes
Fumitremorgin A	Polycyclic aromatic Heterocyclic Hydrocarbon	Indenes
Fumitremorgin B	Polycyclic aromatic Heterocyclic Hydrocarbon	Indenes
Fumitremorgin C	Polycyclic aromatic Heterocyclic Hydrocarbon	Indenes

O-methylparvulenone	Polycyclic aromatic Heterocyclic Hydrocarbon	Napthalenes
Parvulenone	Polycyclic aromatic Heterocyclic Hydrocarbon	Napthalenes
Epi-aculin A	Unclassified	Unclassified
Epiheveadide	Unclassified	Unclassified
Ergosta-7,22-diene-**3β,5α,6β**-triol	Unclassified	Unclassified
Flavipin	Unclassified	Unclassified
Hydroxyaspergillic acid	Unclassified	Unclassified
JBIR-137	Unclassified	Unclassified
JBIR-138	Unclassified	Unclassified
Maltoryzine	Unclassified	Unclassified
Phomaligin A	Unclassified	Unclassified
Radarin	Unclassified	Unclassified
Selenite	Unclassified	Unclassified
Siderin	Unclassified	Unclassified
Sulphinine	Unclassified	Unclassified
**α,β**-**Dehydrocurvularin (α,β**-DC)	Unclassified	Unclassified
**α**-sarcin	Unclassified	Unclassified
